# Orphan response regulator CovR plays positive regulative functions in the survivability and pathogenicity of *Streptococcus suis* serotype 2 isolated from a pig

**DOI:** 10.1186/s12917-023-03808-9

**Published:** 2023-11-22

**Authors:** Yanyan Zhang, Rui Li, Qian Li, Yongwei Zhu, Xiaopei Yang, Di Zhao, Bingbing Zong

**Affiliations:** 1 Hubei Key Laboratory of Animal Nutrition and Feed Science, Engineering Research Center of Feed Protein Resources on Agricultural By-products, Ministry of Education, Wuhan Polytechnic University, Wuhan, 430023 China; 2 Hunan International Scientific and Technological Cooperation Base of Brain Tumor Research, Xiangya Hospital, Central South University, Changsha, China; 3 Wuhan animal disease control center, Wuhan, Hubei China

**Keywords:** *Streptococcus suis* type 2, Orphan response regulator CovR, Pathogenicity, Regulative function, Metabolic pathway

## Abstract

**Background:**

*Streptococcus suis* serotype 2 (*S. suis* 2) is an important zoonotic pathogen. Orphan response regulator CovR plays crucial regulative functions in the survivability and pathogenicity of *S. suis* 2. However, research on the CovR in *S. suis* 2 is limited.

**Results:**

In this study, the regulative functions of CovR in the survivability and pathogenicity were investigated in *S. suis* 2 isolated from a diseased pig. The deletion of CovR significantly weakened the survivability and pathogenicity of *S. suis* 2. Compared with the wild-type strain, Δ*covR* showed slower growth rates and thinner capsular polysaccharides. Moreover, Δ*covR* showed reduced adhesion and invasion to Hep-2 cells as well as anti-phagocytosis and anti-killing ability to 3D4 cells and anti-serum killing ability. In addition, the deletion of CovR significantly reduced the colonisation ability of *S. suis* 2 in mice. The survival rate of mice infected with Δ*covR* was increased by 16.7% compared with that of mice infected with *S. suis* 2. Further, the deletion of CovR led to dramatic changes in metabolism-related pathways in *S. suis* 2, five of those, including fructose and mannose metabolism, glycerolipid metabolism, ABC transporters, amino sugar and nucleotide sugar metabolism and phosphotransferase system, were significantly down-regulated.

**Conclusions:**

Based on the results, CovR plays positive regulative functions in the survivability and pathogenicity of *S. suis* 2 SC19 strain isolated from a pig.

**Supplementary Information:**

The online version contains supplementary material available at 10.1186/s12917-023-03808-9.

## Introduction

*Streptococcus suis* type 2 (*S. suis* 2) is an important zoonosis pathogen that causes huge economic losses to the global pig industry and poses a serious threat to human health [1–4]. A recent study reported that in Chinese pig farms, *S. suis* 2 was the most prevalent bacterial pathogen from 2013 to 2017 [5]. So far, *S. suis* 2, as an emerging zoonotic agent, has infected more than 1,642 people worldwide (as of 2013) and human infected with *S. suis* 2 was reported frequently all over the world [2, 6–8]. These reports above suggested that the threat of *S. suis* 2 to pig industry and people have always existed.

Multiple factors determine the threat of *S. suis* 2 to pigs and humans, mainly including transmission mode, virulence factors and environmental adaptation [9–12]. When *S. suis* 2 spreads to the host, infection occurs in several steps. In the first step, *S. suis* 2 must colonise the host, and in the second step, *S. suis* 2 must be able to survive in the blood after passing through the mucosal layer. Finally, *S. suis* 2 reaches different target tissues through the bloodstream, causing systemic infection and streptococcal toxic shock-like syndrome (STSLS) by inducing pro-inflammatory responses and crossing the blood-brain barrier [9].

To successfully colonise and infect the host, a variety of virulence factors are needed in *S. suis* 2 [13–15]. During host colonisation, several proteinaceous factors, including fibronectin-binding protein, enolases, dipeptidyl-peptidase-4, glyceraldehyde-3-phosphate dehydrogenase and laminin-binding proteins, are involved in the adhesion to cells or components of the extracellular matrix in *S. suis* 2 [13, 16, 17]. Immunoglobulin A1 (IgA1) protease could increase the ability of *S. suis* 2 to cross mucosa membranes by degrading mucosal immunoglobulins A [18]. After colonising the host, the suilysin secreted by *S. suis* 2, which is a haemolysin with cytotoxic properties, can enable *S. suis* 2 to cross the epithelial barrier by lysing the epithelial cells [19]. Subsequently, under the action of various virulence factors, *S. suis* 2 can escape the immune system and enable bacterial dissemination in the bloodstream [9, 20]. After entering the bloodstream, the polysaccharide capsule (CPS) can protect *S. suis* 2 against phagocytosis by monocytes, macrophages, dendritic cells, and neutrophils and help *S. suis* 2 to survive in the cells [20]. In addition, serine proteases can prevent the chemoattraction of phagocytes by depredating the chemokines (Chemokine (C-Cmotif) ligand 5, CCL5 and C-X-C motif chemokine ligand 8, CXCL8) at the infection site [21]. The factor H-binding protein helps *S. suis* 2 against phagocytosis by regulating the alternative pathway of the complement system [22]. Several DNases can assist *S. suis* 2 in escaping the innate immunity of the host by degrading neutrophil extracellular traps [23]. When *S. suis* 2 crosses the mucosal membranes and successfully escapes the immune system, the components presented at the surface of *S. suis* 2 can trigger the pro-inflammatory response of the host by stimulating an excessive activation of T helper cell type 1 (Th1) response, leading to septic shock [13].

These pathogenic factors above are not always expressed all the time in the pathogenesis of *S. suis* 2. Only when needed, *S. suis* 2 will regulate the expression of the corresponding virulence factors according to the specific living environment to improve pathogenicity. Therefore, these regulative factors play important roles in the pathogenesis of *S. suis* 2 [13]. To date, two-component signal transduction systems (TCSs) have been shown to be involved in the modulation of *S. suis* 2 pathogenicity [24]. For example, 15 TCSs have been identified in Chinese *S. suis* 2 strain 05ZYH33 isolated from an infected human brain [25]. Among them, orphan response regulator CovR improves the environmental adaptability of *S. suis* 2 strain 05ZYH33 by regulating the expression levels of multiple virulence factors [25]. So far, the regulative function of CovR has been studied in *S. suis* 2 strain 05ZYH33 isolated from human brain. However, the regulative function of CovR in *S. suis* 2 strain SC19 isolated from a diseased pig has not been studied. Previous studies reported that the CovS/CovR systems in different clinical isolates of *Streptococcus agalactiae* have different regulative functions [26, 27]. For example, the CovS/CovR systems play a negative regulative role in *Streptococcus agalactiae* NEM316 from a neonate blood culture (early onset disease) [26], whereas they played a positive regulative role in *Streptococcus agalactiae* type Ia strain 515 and type V strain 2603 V/R from clinical isolates [27]. This makes it crucial to study the regulative functions of CovR in different clinical isolates to elucidate the pathogenic mechanism of *S. suis* 2.

In this study, the effects of the deletion of CovR on the virulence of *S. suis* 2 SC19 strain, isolated from a diseased pig, were studied. Furthermore, the target genes or signalling pathways regulated by CovR were studied in *S. suis* 2 strain SC19 by RNA-Seq.

## Materials and methods

### Bacteria strains, plasmids and growth conditions

The *S. suis* 2 strain SC19, which was isolated from the brain of a diseased pig, and plasmid pSET4s and pSET2 were supplied by Dr Sekizaki (National Institute of Animal Health, Japan). The *S. suis* 2 strain SC19 was cultured as previously described [28]. Tryptic soy broth (TSB) or tryptic soy agar (TSA) (Difco Laboratories, Detroit, MI, USA) supplemented with 10% newborn bovine serum phage-free (Sijiqing Biological Engineering Materials Co., Ltd., Hangzhou, China) was used to cultivate SC19. Luria-Bertani (LB) broth or LB agar was used to cultivate *Escherichia coli* (*E. coli*) strain DH5α (TaKaRa Biotechnology, Dalian, China) at 37℃. We used 100 µg/mL spectinomycin (Sigma, St Louis, MO, USA) for SC19 and 50 µg/mL spectinomycin for DH5α for the replication of the shuttle vector pSET4s (a thermosensitive suicide vector), which was employed to knockout the gene in SC19.

### Construction of mutant Δ *covR* strain and complementation of *covR* deletion

Mutant Δ*covR* strain was constructed by using the allelic exchange method as previously described, with some modifications [28]. The recombinant plasmid pSET4s::*covR*, including two flanking regions (1,000 bp) of gene *covR*, was electroporated into strain SC19. Subsequently, SC19 with plasmid pSET4s::*covR* was cultured on a TSA plate with 100 µg/mL spectinomycin at 28℃. Under these culture conditions, the plasmid pSET4s::*covR* was inserted into the SC19 genome by homologous recombination at 28℃, and a single-crossover clone deriving from the integration of pSET4S:: *covR* into the genome was generated at 37℃ by spectinomycin selection. Subsequently, a double-crossover clone without pSET4s was generated by culturing the single crossover on TSB or TSA without spectinomycin at 37℃. The mutant Δ*covR* strain was identified by PCR as the mutant or wild-type strain (WT) was generated after plasmid loss. The complementation CΔ*covR* strain was constructed by using *E. coli*-*S. suis* shuttle vector pSET2 as previously described [29, 30].

### Growth characteristics, capsular polysaccharide (CPS) assay and colony morphology

To clarify the effects of the deletion of gene *covR* on the growth characteristics and colony-forming units (CFU), SC19, Δ*covR* and CΔ*covR* at different time points were determined. First, SC19, Δ*covR* and CΔ*covR* were taken out from the freezer (Haier, Qingdao, China) and kept at room temperature. Subsequently, 10 µL of SC19, Δ*covR* or CΔ*covR* was taken and streaked on the plates, which were placed into an incubator (Jing Hong, Shanghai, China) for cultivation at 37℃. Finally, a single colony was picked from the plate and inoculated into TSB. After incubation for 6 h, the cultures were transferred into a new TSB according to 1:100, and the CFUs of SC19, Δ*covR* and CΔ*covR* were measured every 2 h.

To visualise the CPS on the surface of *S. suis* 2, transmission electron microscopy (TEM, HITACHI Transmission Electron Microscope HT7700, Hitachi Limited, Tokyo, Japan) was applied as previously described, with minor modifications [28]. Briefly, 10 mL of the cultured SC19, Δ*covR* and CΔ*covR* (OD_600_ = 0.8) was collected by centrifugation at 6,000 rpm for 10 min at 4℃, and the supernatant was removed. After washing the pellets three times with phosphate-buffered saline (PBS), they were fixed by using PBS with 2.5% glutaraldehyde (Servicebio, Wuhan, China) at room temperature for 2 h. Subsequently, these samples were further processed by Servicebio. The CPS on the surface of *S. suis* 2 was visualised using an HT7700 TEM. The thickness of the CPS on the surface of *S. suis* 2 was determined via Adobe Photoshop (Software version: 22.0.0 20,201,006.r.35 2020/10/06: 4587a1caa63 × 64).

Strains SC19, Δ*covR* and CΔ*covR*, at the exponential growth phase (OD_600_ = 0.8), were used to determine the colony sizes. Briefly, 10 µL of SC19, Δ*covR* and CΔ*covR* were plated on TSA plates after dilution, and the plates were placed in an incubator at 37℃ for 24 h. Subsequently, the diameters of clones of SC19, Δ*covR* and CΔ*covR* on the plates were measured using Adobe Photoshop 2021.

### Cell assays

Human larynx epidermoid carcinoma cells (Hep-2) and porcine alveolar macrophages 3D4/21 (CRL-2843) from pig lung were stored in the laboratory and cultured according to a method described previously [28]. After washing twice with PBS, 400 µL of Hep-2 cells in RPMI 1640 medium (Gibco, New York, USA) with 5% heat-inactivated foetal bovine serum (hiFBS, Gibco, New York, USA) was separately seeded into 24 wells (Corning). Subsequently, 100 µL of *S. suis* 2 (10^7^ CFU/mL) was separately added to 24 wells (MOI = 20), and the plates were incubated with 5% CO_2_ at 37℃ for 2 h. After this, *S. suis* 2 that did not adhere to the cells were washed away with PBS. After a part of cells were lysed by water with 0.025% Triton X-100 (Solarbio, Beijing, China), the number of *S. suis* 2 adhered to the cells was determined by dilution, another part of the cells was lysed after interacting with gentamicin (100 µg/well, Solarbio) and penicillin-G (5 µg/well, Solarbio, Beijing, China) for 1 h to kill extracellular bacteria, and *S. suis* 2 that had invaded the cells were counted by dilution. The phagocytosis and killing of *S. suis* 2 by 3D4 cells were conducted as previously described [28].

### Serum bactericidal test

Cells of *S. suis* 2 at the mid-log phase were collected by centrifugation for 10 min at 6,000 rpm at 4℃. The supernatant was removed, and *S. suis* 2 was washed twice with PBS, followed by dilution to 10^6^ CFU/mL in PBS. Subsequently, 100 µL of *S. suis* 2 was added to 900 µL porcine serum which was not anti-*S. suis* 2 IgG antibody. The mixtures were thoroughly mixed and incubated under gentle shaking at 37℃ for 3 h. The bacterial numbers were determined by serial dilution plating at 0, 1, 2 and 3 h. The survival rate of *S. suis* 2 was equal to (CFU of 0, 1, 2 or 3 h)/(CFU of 0 h)*100%.

### Animal experiments

The animal experiments were approved by the Animal Care and Use Committee and conducted according to the guidelines of the Research Ethics Committee of the College of Animal Science and Nutritional Engineering at Wuhan Polytechnic University (No. WPU202206002). In order to give maximum welfare to the experimental mice, experimental mice were anesthetized with intraperitoneally injection of 50 µg/kg Zoletil Zoletil 50 (Zoletil 50, Virbac, France). To investigate the effects of the deletion of *covR* on the pathogenesis of *S. suis* 2, 51 5-week-old female BALB/c mice, purchased from the Wuhan Center for Disease Prevention & Control (Wuhan, China), were infected intraperitoneally with *S. suis* 2. To assess the effects of *covR* deletion on virulence of *S. suis* 2, 36 mice were randomly divided into three groups of 12 mice each (PBS, SC19 and Δ*covR*). Cells of SC19 and Δ*covR* were collected in the mid-log phase and resuspended to 1 × 10^9^ CFU/mL in PBS. Mice in the SC19 group were injected intraperitoneally with SC19 (200 µL per mouse), mice in the Δ*covR* group were injected intraperitoneally with Δ*covR* (200 µL per mouse), and mice in the PBS group were injected intraperitoneally with PBS (200 µL per mouse). The survival rates of mice in the different groups were monitored every 12 h from 0 to 120 h after infection. After 120 h, the surviving mice were anaesthetized with intraperitoneally injection of 50 µg/kg Zoletil Zoletil 50 (Zoletil 50, Virbac, France), subsequently, mice were euthanized by cervical dislocation. To determine the effects of gene *covR* deletion on the ability of *S. suis* 2 to colonise the host, 15 mice were randomly divided into three groups of five mice each (PBS, SC19 and Δ*covR*). Cells of SC19 and Δ*covR* were collected in the mid-log phase and resuspended to 1 × 10^8^ CFU/mL in PBS. Mice in the SC19 group were injected intraperitoneally with SC19 (200 µL per mouse), mice in Δ*covR* group were injected intraperitoneally with Δ*covR* (200 µL per mouse), and mice in PBS group were injected intraperitoneally with PBS (200 µL per mouse). At 10 h post infection, the bacterial loads in the blood, heart, liver, kidney, spleen and lung of mice in the different groups were determined as previously described [3].

### RNA-Seq and quantitative real-time polymerase chain reaction (qRT-PCR)

To determine the effect of gene *covR* on the mRNA levels of genes in *S. suis* 2, the mRNA levels of genes in SC19 and Δ*covR* were determined by RNA-Seq. The *S. suis* 2 SC19 chromosome, the complete genome (GenBank: CP020863.1), was used as reference genome in this study. Briefly, 20 mL of SC19 and Δ*covR*, cultivated to the exponential growth phase, was centrifuged separately at 6,000 rpm for 10 min at 4℃, and the collected SC19 and Δ*covR* cells were placed in dry ice and transported to Majorbio (Shanghai, China) for total RNA extraction, sequencing and library construction. The qRT-PCR was conducted as previously described [28]. The primers listed in Table S 1 were designed according to the genomic sequence of SC19 and used for qRT-PCR.

### Statistical analysis

All statistical analyses were performed in GraphPad Prism 9.0.0 (San Diego, USA). Survival data were analysed with the log-rank (Mantel-Cox) test. The two-tailed Mann–Whitney test was used to analyse differences in bacterial burdens, and the two-tailed unpaired t test was employed to analyse the growth rate and thickness of CPS and bacterial survival in cells; *P*-values < 0.05 were considered statistically significant. The data including differentially expressed genes (|Log2(Fold change)| > 0 and Padj < 0.05), functional annotation of differentially expressed genes (GO and KEGG annotation) and functional enrichment analysis of differentially expressed genes (GO and KEGG enrichment, false discovery rate (FDR) < 0.05) were analysed on the Majorbio Cloud Platform with default parameters (www.majorbio.com).

## Results

### Effects of the deletion of *covR* on CPS thickness, colony size and growth of *S. suis *2 SC19

**Fig. 1:**
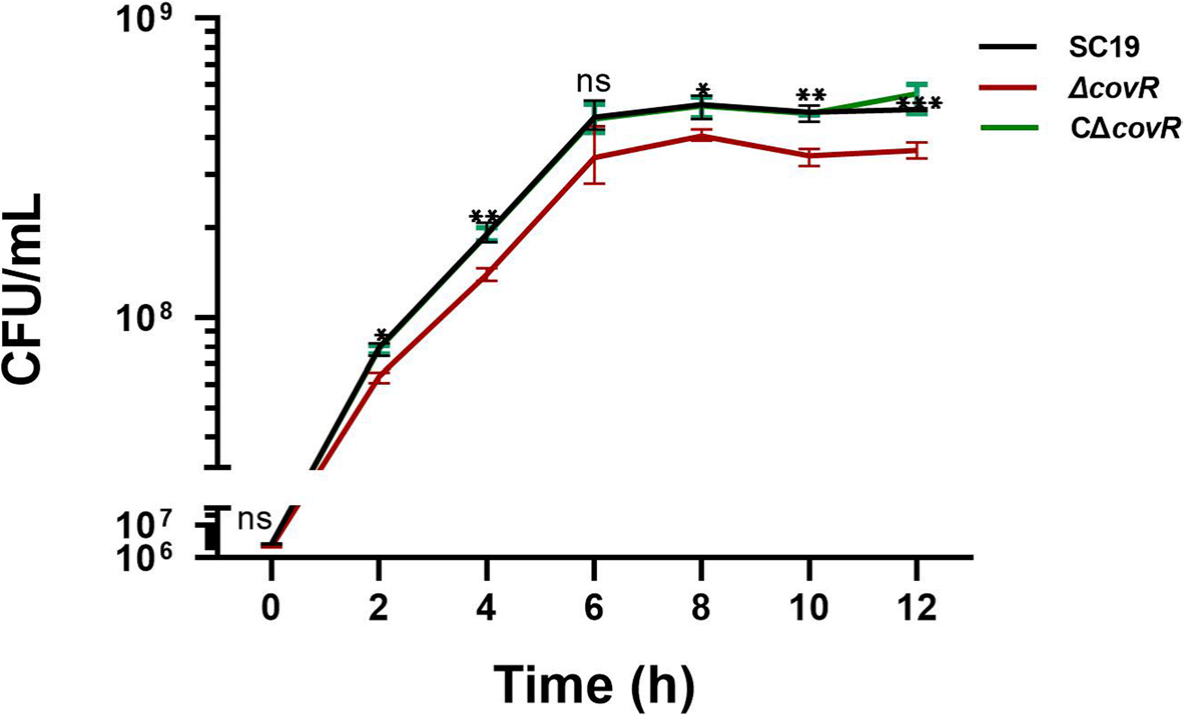
Effects of CovR deletion on the growth characteristics of *S. suis* 2. Data are means ± SD from three biological replicates. Statistical analyses were performed by using the two-tailed unpaired t test. Statistically significant differences are indicated. *P < 0.05, **P < 0.01, ***P < 0.001

**Fig. 2:**
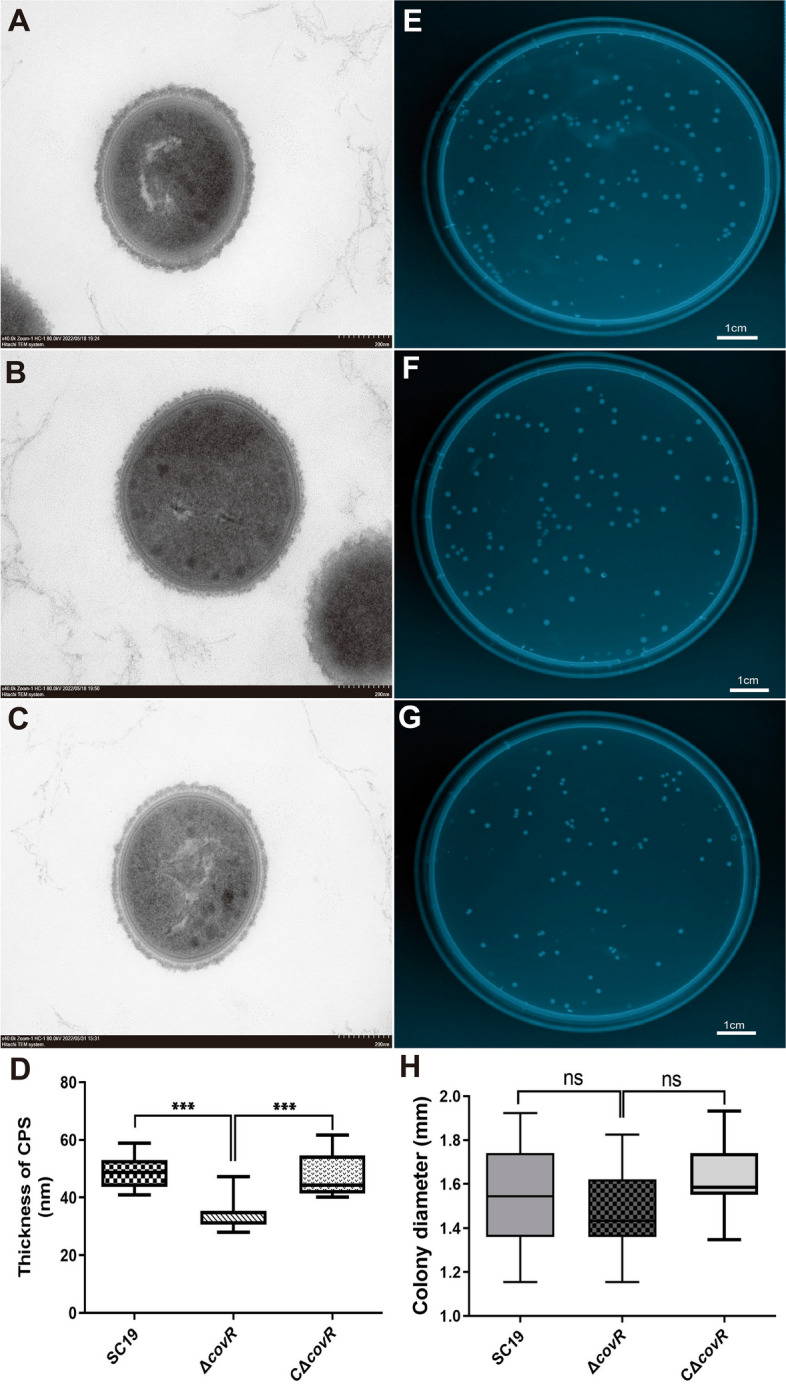
Effects of CovR deletion on the characteristics of *S. suis* 2. **A** CPS on the surface of *S. suis* 2 SC19. **B** CPS on the surface of Δ*covR*. **C** CPS on the surface of CΔ*covR*. **D** CPS thickness of SC19, Δ*covR* and CΔ*covR*. **E** Colonies of SC19. **F** Colonies of Δ*covR*. **G** Colonies of CΔ*covR*. **H** Colony diameters of SC19, Δ*covR* and CΔ*covR*. Data are means ± SD from three biological replicates. Scale bar: 200 nm (**A**, **B** and **C**). Scaler bar: 1 cm (**E**, **F** and **G**). Statistical analyses were performed by using the two-tailed unpaired t test. Statistically significant differences are indicated. *P < 0.05, **P < 0.01, ***P < 0.001

The mutant Δ*covR* and the complementation CΔ*covR* strains were successfully constructed (Fig. S 1). Gene *covR* deletion significantly weakened the growth rate of SC19, and the growth rate of Δ*covR* was significantly reduced from 2 h compared with that of SC19. The CΔ*covR* grew as well as SC19 (Fig. 1). The Δ*covR* displayed a thinner CPS compared with that of SC19 (Fig. 2A, B, D). The thickness of CPS on the surface of SC19 ranged between 40 and 60 nm, whereas the thickness of CPS on the surface of Δ*covR* ranged from 25 to 40 nm (Fig. 2D). Gene *covR* deletion significantly reduced the thickness of CPS on the surface of *S. suis* 2, whereas the thickness of CPS on the surface of CΔ*covR* was similar to that of SC19 (Fig. 2A, C, D). Gene *covR* deletion did not affect the average diameter of the SC19 colony (Fig. 2E, F, G, H).

### Effects of the deletion of *covR* on the adhesion and invasiveness of *S. suis *2 SC19

**Fig. 3:**
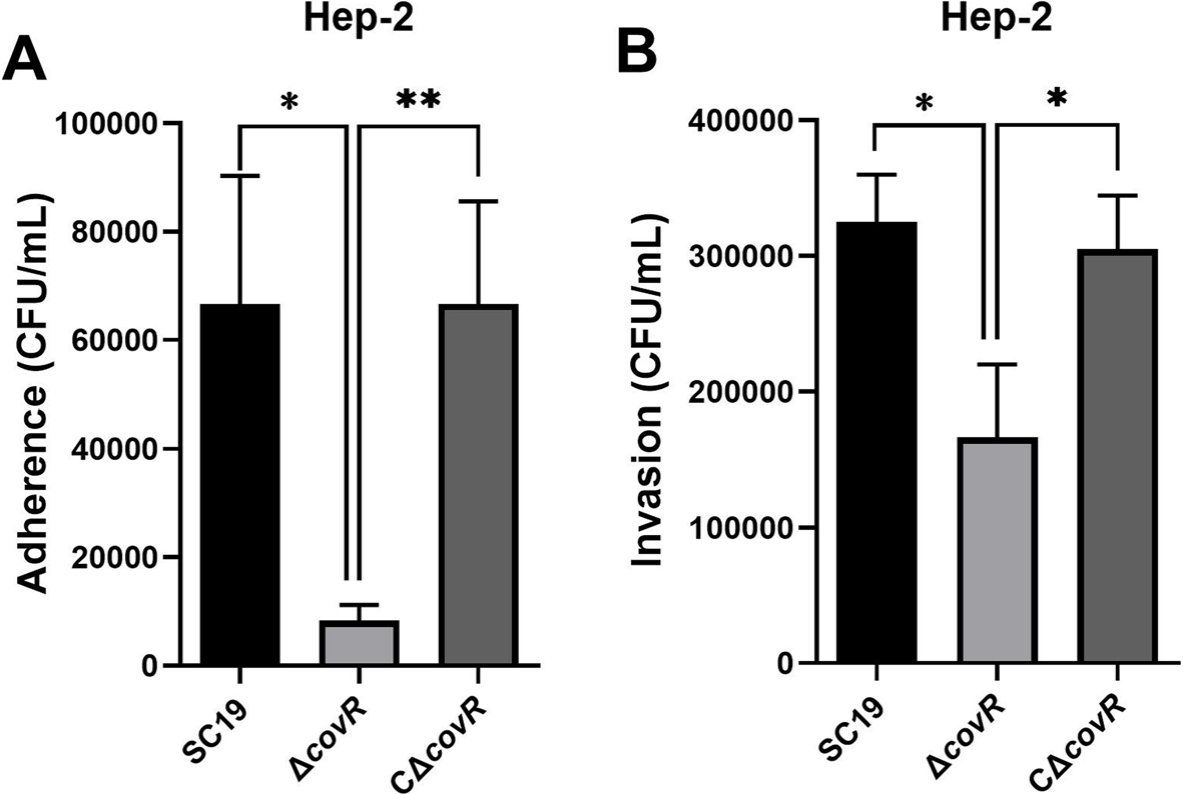
Effects of CovR deletion on *S. suis* 2 adhesion and invasion. **A** Adhesion of SC19, Δ*covR* and CΔ*covR* to Hep-2 cells. **B** Invasion capacity of SC19, Δ*covR* and CΔ*covR* for Hep-2 cells. The results are expressed as means ± SD from three biological replicates. Statistical analyses were performed by using the two-tailed unpaired t test. Statistically significant differences are indicated. *P < 0.05, **P < 0.01

The effects of gene *covR* deletion on the ability of *S. suis* 2 SC19 to adhere to and invade Hep-2 cells were investigated in vitro. The Δ*covR* displayed reduced adhesion and invasion to Hep-2 cells compared with *S. suis* 2 SC19. The adhesion of Δ*covR* to Hep-2 cells was approximately 0.15-fold compared with that of *S. suis* 2 SC19 (Fig. 3A), and the invasion of Δ*covR* to Hep-2 cells was approximately 0.51-fold compared with that of *S. suis* 2 SC19 (Fig. 3B). The adhesion and invasion abilities of CΔ*covR* were similar to those of SC19 (Fig. 3AB).

### Effects of the deletion of *covR* on the anti-phagocytic, anti-killing and anti-serum activities of *S. suis *2 SC19

**Fig. 4:**
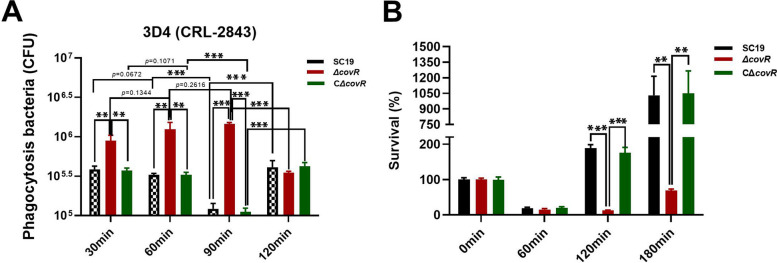
Ability of *S. suis* 2 to escape from the innate immune system. **A** Numbers of SC19, Δ*covR* and CΔ*covR* phagocytosed by 3D4 cells at 37℃ for 30, 60, 90 and 120 min. **B** Numbers of viable SC19 Δ*covR* and CΔ*covR* in pig serum at 37℃ for 0, 60, 90 and 180 min. Data are expressed in means ± SD from three biological replicates. Statistical analyses were performed by using the two-tailed unpaired t test. Statistically significant differences are indicated. **P < 0.01, ***P < 0.001

To study the effects of gene *covR* deletion on the interactions between *S. suis* 2 SC19 and phagocytosis, the anti-phagocytic and anti-killing activities of Δ*covR* were investigated and compared with those of *S. suis* 2 SC19. The numbers of Δ*covR* cells phagocytosed by 3D4CRL-2843 were higher than those of *S. suis* 2 SC19 and CΔ*covR* at 30, 60 and 90 min (Fig. 4A). Moreover, the number of phagocytosed Δ*covR* cells tended to increase from 30 to 90 min, whereas those of phagocytosed *S. suis* 2 SC19 and CΔ*covR* were continuously reduced from 30 to 90 min (Fig. 4A). However, the numbers of *S. suis* 2 SC19 and CΔ*covR* cells in 3D4CRL-2843 at 120 min were significantly increased compared with those of *S. suis* 2 SC19 and CΔ*covR* in 3D4CRL-2843 at 90 min, and the number of Δ*covR* in 3D4CRL-2843 at 120 min was significantly reduced compared with that of Δ*covR* in 3D4CRL-2843 at 90 min. These results suggest that the anti-phagocytic and anti-killing abilities of Δ*covR* were significantly weakened compared with those of *S. suis* 2 SC19 due to gene *covR* deletion (Fig. 4A). The anti-phagocytic and anti-killing abilities of CΔ*covR* were similar to those of SC19 (Fig. 4A).

To investigate the effects of gene *covR* deletion on the anti-serum bactericidal ability of *S. suis* 2 SC19, the interactions between *S. suis* 2 SC19 and porcine serum were studied. The numbers of *S. suis* 2 SC19 and Δ*covR* cells at 60 min were significantly reduced compared with those of *S. suis* 2 SC19 and Δ*covR* cells at 0 min. The numbers of *S. suis* 2 SC19 and Δ*covR* cells were lowest during this period, and the number of Δ*covR* cells was similar to that of *S. suis* 2 SC19 in porcine serum at 60 min. After incubation for 120 min, the numbers of *S. suis* 2 SC19 and CΔ*covR* cells continuously increased, and those of *S. suis* 2 SC19 and CΔ*covR* cells at 120 and 180 min were significantly higher than those of *S. suis* 2 SC19 and CΔ*covR* at 0 min. Moreover, the number of *S. suis* 2 SC19 cells was significantly higher than that of Δ*covR* cells at 120 and 180 min. These results suggest that the anti-serum bactericidal ability of Δ*covR* was significantly reduced compared with that of SC19 due to gene *covR* deletion (Fig. 4B). The anti-serum bactericidal ability of CΔ*covR* was similar to that of SC19 (Fig. 4B).

### Effects of the deletion of *covR* on the virulence of *S. suis *2 SC19

The phenotypes of the complemented mutant CΔ*covR* were restored in terms of growth rate, capsule thickness, adhesion and invasion, anti-phagocytic and anti-killing as well as anti-serum bactericidal ability, indicating that these phenotypic changes were caused by *covR* gene deletion. Therefore, the complemented mutant was not used in the animal experiments.

**Fig. 5:**
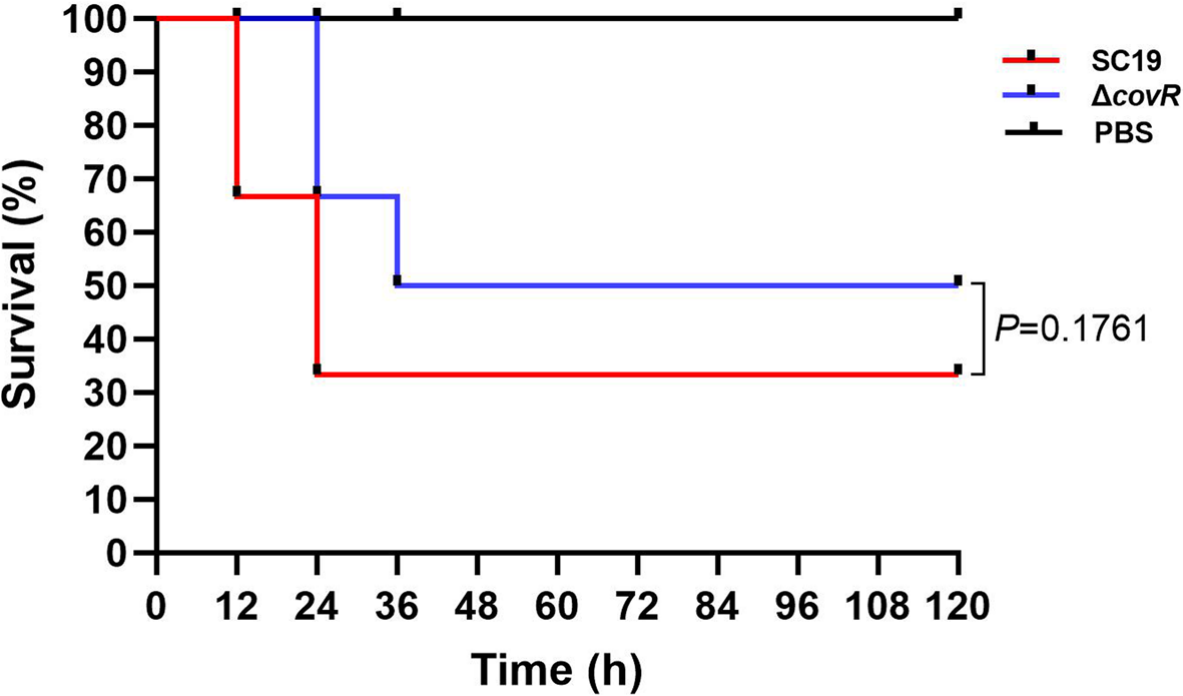
Effect of CovR deletion on the survival rate of mice infected with *S. suis* 2, 12 mice per group. Survival data were analysed with the log-rank test

To investigate the effect of gene *covR* deletion on the virulence of *S. suis* 2 SC19, 36 BALB/c mice in different groups were injected intraperitoneally with SC19, Δ*covR* (2.0 × 10^8^ CFU) or PBS. The experiment was terminated when no mice died for 84 consecutive hours during the experiment. At 0 to 12 h after infection, the survival rate of mice infected with SC19 was 66.6%, and at 12 to 24 h after infection, the survival rate of mice infected with SC19 was 33.3%. At 24 h after infection, no deaths occurred in the SC19 group. At 0 to 24 h after infection, the survival rate of mice infected with Δ*covR* was 66.6%, and at 24 to 36 h after infection, the survival rate of mice infected with Δ*covR* was 50%; at 36 h after infection, no deaths occurred in the Δ*covR* group (Fig. 5). Although no significant difference was observed between the SC19 group and the Δ*covR* group, the survival rate of mice infected with Δ*covR* was 16.7% higher (Fig. 5). Based on these results, mice infected with Δ*covR* had an increased survival rate and delayed death, suggesting that gene *covR* deletion reduced the virulence of *S. suis* 2 SC19.

### Effects of the deletion of *covR* on the colonisation ability and pathogenicity of *S. suis *2 SC19

**Fig. 6:**
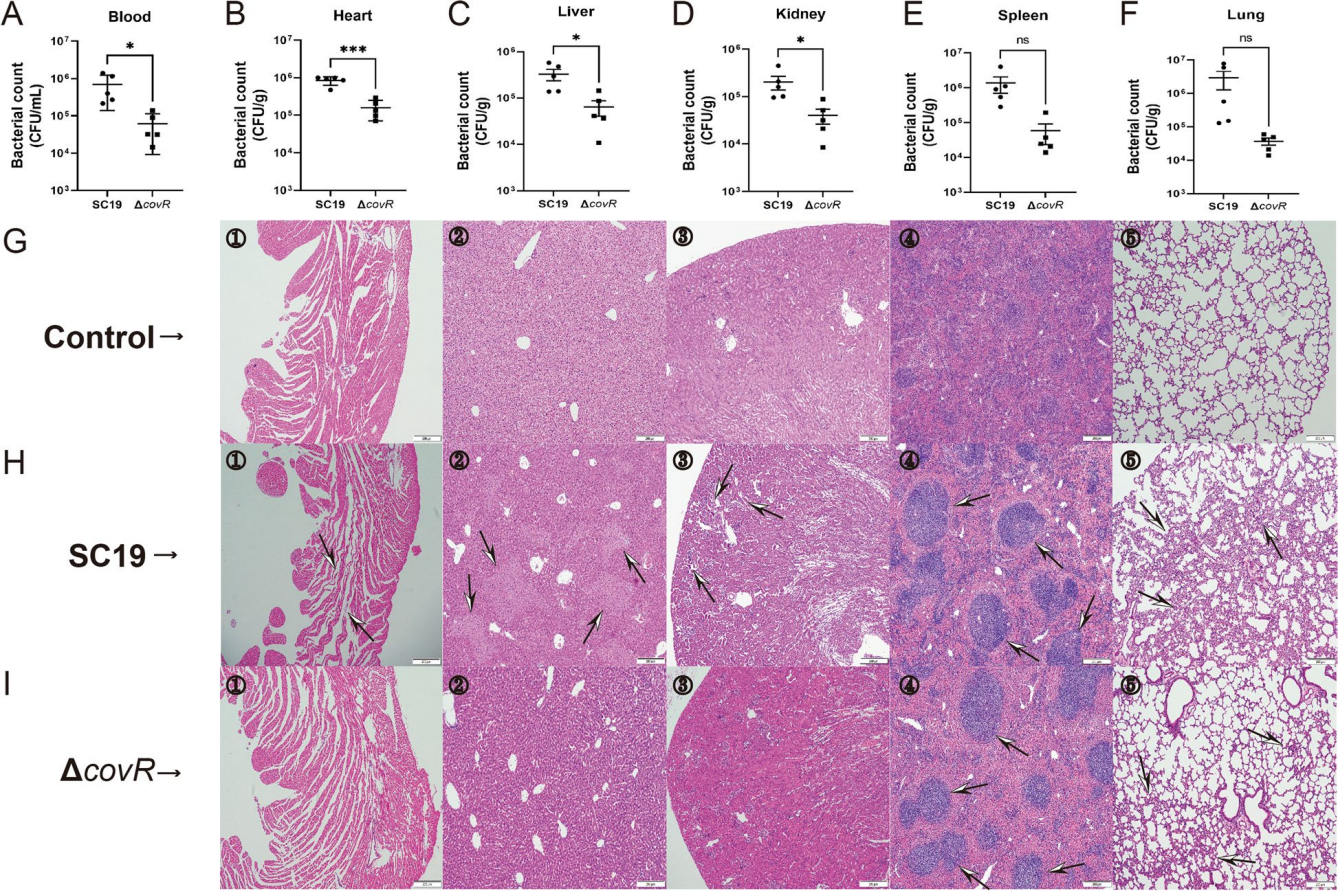
Effect of CovR deletion on the pathogenicity of *S. suis* 2, five mice per group. Numbers of SC19 and Δ*covR* cells in the blood (**A**), heart (**B**), liver (**C**), kidney (**D**), spleen (**E**) and lung (**F**) of mice at 10 h post infection. Pathological examination of the blood (**①**), heart (**②**), liver (**③**), kidney (**④**), spleen (**⑤**) and lung (**⑥**) of mice. **G** Control, **H** infected with SC19, **I** infected with Δ*covR*. Scaler bar: 200 μm (H&E). Data are means ± SD. Statistical analyses were performed by using the two-tailed Mann-Whitney test. Statistically significant differences are indicated. *P < 0.05, ***P < 0.001

To study the effect of gene *covR* deletion on the colonisation ability of *S. suis* 2 SC19, the number of Δ*covR* cells was determined and compared with those of *S. suis* 2 SC19 in blood, heart, liver, kidney, spleen and lung of mice infected with 2 × 10^7^ CFU SC19 or Δ*covR*. Dramatic differences in the numbers between *S. suis* 2 SC19 and Δ*covR* in blood, heart, liver and kidney were observed at 8 h post infection. The number of Δ*covR* cells was significantly lower than that of *S. suis* 2 SC19 in the blood (Fig. 6A), heart (Fig. 6B), liver (Fig. 6C) and kidney (Fig. 6D). Although there were less Δ*covR* than *S. suis* 2 SC19 cells in the spleen (Fig. 6E) and lung (Fig. 6F), this difference was not significant. These results suggest that the colonisation ability of Δ*covR* was significantly reduced compared with that of SC19 due to gene *covR* deletion.

To compare the pathogenicity of *S. suis* 2 SC19 and Δ*covR*, mice were separately infected intraperitoneally with 2 × 10^7^ CFU SC19 or Δ*covR*. At 8 h post infection, analyses of the heart, liver, kidney, spleen and lung from mice exhibited severe pathological damage. After *S. suis* 2 SC19 infection, cardiomyocytes showed obvious shrinkage, separation and damage (Fig. 6H-①), the liver showed significant fatty infiltration (Fig. 6H-②), the cells of the renal cortex were atrophied and the intercellular space was enlarged (Fig. 6H-③), the germinal centre of the kidney was larger and more mature (Fig. 6H-④), and there were numerous inflammatory cells in the lung bronchus (Fig. 6H-⑤). Kidneys with bleeding spots and spleen enlargement and congestion were also found. Compared with the pathological damage in mice caused by SC19, that in mice caused by Δ*covR* was significantly reduced. The Δ*covR* infection caused mild shrinkage, separation and damage of cardiomyocytes in mice (Fig. 6I-①), fatty infiltration was not observed in the liver of mice infected with Δ*covR*(Fig. 6I-②), the cells of the renal cortex were not atrophied, and the intercellular space was not enlarged in the kidneys of mice infected with Δ*covR*(Fig. 6I-③). The infiltration of inflammatory cells was significantly reduced in the pulmonary bronchus (Fig. 6I-⑤) of mice infected with Δ*covR*. In accordance with the pathological changes in mice kidneys caused by SC19 infection, Δ*covR* infection also caused that the germinal centre of the kidney became larger and more mature (Fig. 6I-④). These results suggest that the pathogenicity of Δ*covR* was significantly reduced compared with that of SC19 due to gene *covR* deletion.

### Changes in mRNA levels of genes in *S. suis *2 SC19 after *covR* deletion

Based on the effects of gene *covR* deletion on the survivability and pathogenicity of *S. suis* 2, gene *covR* deletion significantly reduced the growth, resistance to phagocytosis and pathogenicity of *S. suis* 2. Gene *covR*, as a two-component regulative system, plays an important regulative role in the survivability and pathogenicity of *S. suis* 2. To further determine which genes or pathways affect the survivability and pathogenicity of *S. suis* 2, genes differently expressed between *S. suis* 2 SC19 and Δ*covR* were mined by RNA-Seq, and 1,836 genes were identified in the SC19 and Δ*covR* groups. The number of genes shared between the two groups was 1,823 (Fig. S 2A). The differences between SC19 and Δ*covR* groups were calculated based on principal components analysis (PCA). The Δ*covR* group was separated from the SC19 groups in the PCA (Fig. S 2B). The expressed genes with |Log2 (Fold change)| > 0 and Padj < 0.05 shared between *S. suis* 2 SC19 and Δ*covR* were considered to be significantly different. The analysis results showed that 114 genes (Table S 2) were significantly down-regulated, and 117 genes (Table S 3) were significantly up-regulated after gene *covR* deletion based on RNA-Seq (Fig. S 3). This suggests that gene *covR* could regulate the survivability and pathogenicity of *S. suis* 2 by down-regulating 114 genes and up-regulating 117 ones.

### Metabolic pathway analysis of the genes differentially expressed between *S. suis* 2 SC19 and Δ*covR*

**Fig. 7:**
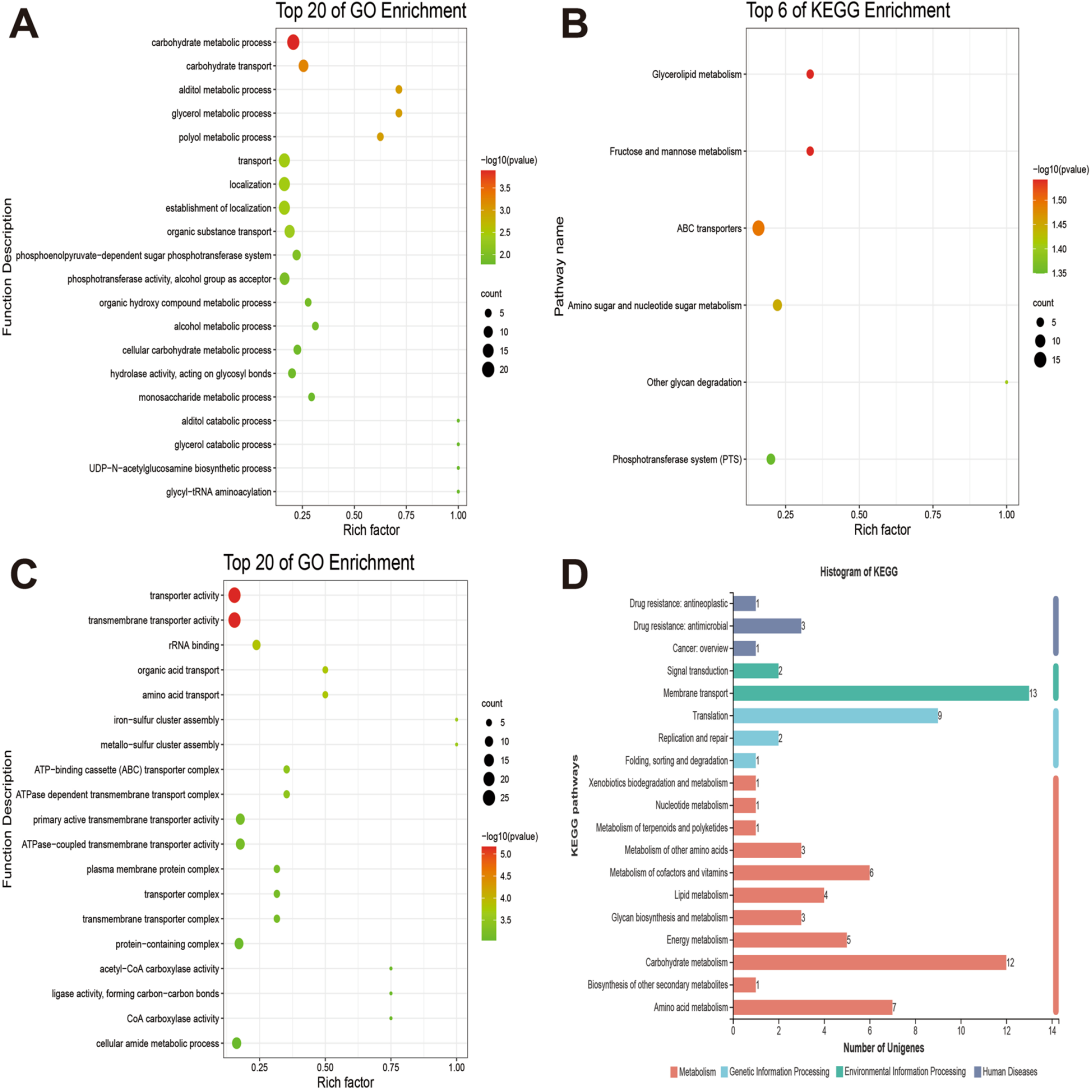
GO and KEGG analyses of 114 down-regulated genes (**A** and **B**). **A** GO enrichment analysis. **B** KEGG enrichment analysis. GO and KEGG analysis of 117 up-regulated Genes (**C** and **D**). **C** GO enrichment analysis. **D** KEGG analysis

We performed GO analysis of the differentially expressed genes to annotate the 114 down-regulated genes. Phosphoenolpyruvate-dependent sugar phosphotransferase system, carbohydrate metabolic process, transmembrane transport, carbohydrate transport, regulation of transcription, DNA-templated, DNA replication and glycerol metabolic process were annotated to biological processes. Integral component of the membrane, plasma membrane, cytoplasm, ATP-binding cassette (ABC) transporter complex and extracellular region were annotated to cellular components, and ATP binding, DNA binding, metal ion binding, hydrolase activity, ATPase-coupled transmembrane transporter activity, protein-N(PI)-phosphohistidine-sugar phosphotransferase activity, zinc ion binding and channel activity were annotated to molecular functions (Fig. S 4A). The gene ontology (GO) enrichment analysis results (FDR < 0.05) showed that biological processes, cellular components and molecular functions related to 114 down-regulated genes were significantly different (Fig. 7A). Kyoto Encyclopedia of Genes and Genomes (KEGG) pathway analysis of differential expression genes was performed to annotate the 114 down-regulated genes. Amino acid metabolism, carbohydrate metabolism, energy metabolism, glycan biosynthesis and metabolism, lipid metabolism, metabolism of cofactors and vitamins, metabolism of other amino acids, nucleotide metabolism and xenobiotics biodegradation and metabolism were annotated to metabolism pathways. Folding, sorting, degradation and translation were annotated to genetic information processing. Membrane transport and signal transduction were annotated to environmental information processing. Cellular community-prokaryotes and transport and catabolism were annotated to cellular processes, and aging, endocrine system and environmental adaptation were annotated to organismal systems. Cancer: overview, drug resistance: antimicrobial and infectious disease: bacterial were annotated to human diseases (Fig. S 4B). The pathways screened with a threshold of FDR < 0.05 were considered to be significantly different gene enrichment pathways. The KEGG enrichment pathway results showed that the pathways of glycerolipid metabolism, fructose and mannose metabolism, ABC transporters, amino sugar and nucleotide sugar metabolism, other glycan degradation and phosphotransferase system (PTS) were significantly different (Fig. 7B). The qRT-PCR results (Table S 4) showed that the down-regulated differentially expressed genes (Table 1) related to fructose and mannose metabolism, glycerolipid metabolism, ABC transporters, amino sugar and nucleotide sugar metabolism and phosphotransferase system (PTS) were reliable.

**Table 1:** The genes in the KEGG enrichment pathways for the down-regulated genes

Gene ID	Gene Name	KO ID	KO Name	Fold change
Fructose and mannose metabolism				
B9H01_RS08575	B9H01_RS08575	K02795	manY	0.79238
B9H01_RS04035	pfkB	K00882	fruK	0.877493
B9H01_RS04040	B9H01_RS04040	K02770	fruA	0.819678
B9H01_RS08580	B9H01_RS08580	K02796	manZ	0.832418
**Glycerolipid metabolism**				
B9H01_RS07520	B9H01_RS07520	K07407	galA	0.666382
B9H01_RS03575	glpK	K00864	glpK	0.85005
**ABC transporters**				
B9H01_RS09200	B9H01_RS09200	K17319	lplB	0.682764
B9H01_RS09195	B9H01_RS09195	K17320	lplC	0.694722357
B9H01_RS09190	B9H01_RS09190	K17318	lplA	0.747241755
B9H01_RS10010	B9H01_RS10010	K11710	troB	0.799013897
B9H01_RS10265	B9H01_RS10265	K15772	ganQ	0.843427031
B9H01_RS10260	B9H01_RS10260	K15771	ganP	0.8343401
B9H01_RS10025	B9H01_RS10025	K11707	troA	0.76620194
B9H01_RS10000	B9H01_RS10000	K11709	troD	0.863698938
B9H01_RS10255	B9H01_RS10255	K15770	cycB	0.830055652
B9H01_RS10005	B9H01_RS10005	K11708	troC	0.802254795
**Amino sugar and nucleotide sugar metabolism**				
B9H01_RS08575	B9H01_RS08575	K02795	manY	0.792380203
B9H01_RS06090	nagA	K01443	nagA	0.816709683
B9H01_RS01845	B9H01_RS01845	K00849	galK	0.764379039
B9H01_RS01850	galT	K00965	galT	0.819724823
B9H01_RS08580	B9H01_RS08580	K02796	manZ	0.832418165
**Phosphotransferase system**				
B9H01_RS08575	B9H01_RS08575	K02795	manY	0.792380203
B9H01_RS01065	B9H01_RS01065	K03475	ulaA	0.720561893
B9H01_RS04035	pfkB	K00882	fruK	0.87749257
B9H01_RS01165	B9H01_RS01165	K02761	celB	0.749690724
B9H01_RS04040	B9H01_RS04040	K02770	fruA	0.819677837
B9H01_RS08580	B9H01_RS08580	K02796	manZ	0.832418165
B9H01_RS09285	B9H01_RS09285	K02810	scrA	0.800577967

The GO analysis of differentially expressed genes was used to annotate the 117 up-regulated genes. Translation, amino acid transport, cell division and DNA recombination were annotated to biological processes. Integral component of membrane, cytoplasm, ATP-binding cassette (ABC) transporter complex, plasma membrane and ribosome were annotated to cellular components. ATP binding, ATPase-coupled transmembrane transporter activity, DNA binding, rRNA binding, hydrolase activity, structural constituent of ribosome, transmembrane transporter activity, metal ion binding, oxidoreductase activity, ABC-type amino acid transporter activity and ATP hydrolysis activity were annotated to molecular functions (Fig. S 5). The GO enrichment analysis results (FDR < 0.05) showed that biological processes, cellular components and molecular functions related to 117 up-regulated genes were significantly different (Fig. 7C). The KEGG pathway analysis of differential expression genes was employed to annotate the 117 up-regulated genes. Amino acid metabolism, biosynthesis of other secondary metabolites, carbohydrate metabolism, energy metabolism, glycan biosynthesis and metabolism, lipid metabolism, metabolism of cofactors and vitamins, metabolism of other amino acids, metabolism of terpenoids and polyketides, nucleotide metabolism and xenobiotics biodegradation and metabolism were annotated to metabolism pathways. Folding, sorting and degradation, replication and repair and translation were annotated to genetic information processing. Membrane transport and signal transduction were annotated to environmental information processing. Cancer: overview, drug resistance: antimicrobial and drug resistance: antineoplastic were annotated to human diseases (Fig. 7D). However, the KEGG enrichment pathway results showed that no significantly different pathways related to the up-regulated genes were found. This suggests that gene *covR* deletion caused that these pathways, including glycerolipid metabolism, fructose and mannose metabolism, ABC transporters, amino sugar and nucleotide sugar metabolism, other glycan degradation and phosphotransferase system (PTS), in *S. suis* 2 were significantly different. Moreover, these pathways may play important roles in the survivability and pathogenicity of *S. suis* 2.

## Discussion

Strain *S. suis* 2, as an emerging zoonotic agent, can rapidly sense and adapt to the changing environmental conditions encountered during the invasion and colonisation process by using the TCSs [31, 32]. Previous studies have shown that 15 TCSs that play important roles in bacterial adaptation and the production of bacterial pathogenic factors are encoded in *S. suis* 2 [13]. Of these, CovR is one of the most important one, but respective studies are limited in *S. suis* 2 [25]. Unlike other TCSs that consist of a membrane-embedded sensor kinase and response regulator, the sensor kinase corresponding to response regulator CovR is not found in *S. suis* 2 [33, 34]. Therefore, CovR, as an orphan response regulator, was studied in *S. suis* 2 [25]. A previous study reported that the same regulative systems in the same bacterial genus may evolve different regulatory mechanisms during invasion and colonisation [26]. Therefore, research on the regulative mechanism of CovR in *S. suis* 2 isolated from different hosts will help to reveal the pathogenic mechanism and better understand the evolutionary mechanism of *S. suis* 2, providing a theoretical basis for preventing bacterial transmission and infection.

Previous research has shown that the deletion of CovR leads to a lower growth rate in the Δ*covR* mutant than that in the parent *S. suis* 2 05ZYH33 strain [25]. In agreement with the above finding, our results also show that the deletion of CovR reduced the growth rate of the *S. suis* 2 SC19 strain, and CΔ*covR* grew as well as SC19 (Fig. 1). In addition to rapidly proliferating in new hosts, *S. suis* 2 cells also need to adhere to epithelial cells to avoid mechanical clearance by physiological responses such as coughing and villi movement. Our results showed that the deletion of CovR weakened the abilities of *S. suis* 2 SC19 to adhere to and invade Hep-2 cells, and the adhesion and invasion abilities of CΔ*covR* were similar to those of SC19 (Fig. 3AB). These results suggest that CovR plays an important positive regulative role in the process of *S. suis* 2 crossing the epithelial cell barrier. Our results are, however, not consistent with those of Pan et al. [25], who reported that the deletion of CovR increased the ability of *S. suis* 2 05ZYH33 to adhere to Hep-2 cells. These differences were likely due to differences in the primary mode of infection of 05ZYH33 and SC19 strains. Strain *S. suis* 2 05ZYH33 may infect humans primarily through wounds and hardly crosses the epithelial cell barrier. However, *S. suis* 2 SC19 may infect pigs primarily through the epithelial barrier.

The CPS not only assists *S. suis* 2 in avoiding phagocytosis and killing by phagocytes but also inhibit the activation of the complement system [35–37]. Therefore, CPS is one of the most effective factors for *S. suis* 2 to resist the innate immune response [35]. However, the deletion of CovR not only resulted in a reduced CPS (Fig. 2), but also caused that the anti-phagocytic and anti-killing and anti-serum bactericidal abilities of S. suis 2 SC19 stain were significantly decreased (Fig. 4). The thickness of the CPS of CΔ*covR*, anti-phagocytic and anti-killing ability of CΔcovR, and anti-serum bactericidal abilities of CΔ*covR* were as well as SC19 (Fig. 2ACD and 4AB). The increase in the number of phagocytosed *S. suis* 2 SC19 can be explained as follows: *S. suis* 2 SC19 cells, which were engulfed, were wrapped in phagocytic vesicles, and the autophagosomes formed by the fusion of phagocytic vesicles and lysosomes had bactericidal function. According to the results of the phagocytosis of *S. suis* 2 SC19 by 3D4 cells, we speculate that *S. suis* SC19/CΔ*covR* cells engulfed by 3D4 cells were killed via autophagy at 60–90 min. However, *S. suis* 2 SC19 cells have anti-killing activity; they secrete a haemolysin, which is a pore-forming toxin and can punch holes in the membrane. This leads us to the assumption that the phagocytic *S. suis* 2 SC19 cells were largely killed by autophagosomes at 90 min, but over time, the haemolysin secreted by *S. suis* 2 SC19 formed a numerous pores on the autophagosome membrane at 90 min after *S. suis* 2 SC19 cells had been engulfed. Therefore, the autophagosomes were destroyed, and subsequently, *S. suis* 2 SC19 started to proliferate, which explains its increased cell number at 120 min. However, the deletion of CovR led to a decrease in the ability of Δ*covR* to escape autophagy lysosomal killing, and thus, the number of phagocytosed Δ*covR* was reduced after 120 min. These results, including the thickness of CPS and the adhesion ability, were inconsistent with those of previous studies in *S. suis* 2 05ZYH33, Group A *Streptococcus* and *Streptococcus mutans* [25, 28, 38, 39] and can be explained by the fact that *S. suis* 2 SC19 and the reported strains (*S. suis* 2 05ZYH33, Group A *Streptococcus* and *Streptococcus mutans*) were isolated from different hosts [25, 28, 38, 39]. In addition, the genome comparison results showed that the genomes of *S. suis* 2 SC19 and 05ZYH33 strains were different (Fig. S 6), and there were 128 areas (identity < 85%) and 9 areas (identity < 80%) between the genomes of *S. suis* 2 SC19 and 05ZYH33 strains (Table S 5). Moreover, during long-term interaction with the host, bacteria may evolve specific regulative mechanisms according to the host environment. In previous studies, CovR or CovR/S played negative regulative functions in the pathogenesis of *S. suis* 2 05ZYH33, Group A S*treptococcus* and *Streptococcus mutans* isolated from humans [25, 38, 39]. However, CovR may play a positive regulative function in the pathogenesis of *S. suis* 2 SC19 strain isolated from a diseased pig.

To further verify the positive regulative function of CovR in *S. suis* 2 SC19 strain, the effects of the deletion of CovR on SC19 virulence and colonisation in mice were studied. Consistent with the results above, the deletion of CovR led to a significant reduction in bacterial virulence (Fig. 5) and colonisation ability (Fig. 6), which was most likely due to the reduced abilities of SC19 to adhere to and invade epithelial cells and escape the host’s innate immune response due to CovR deletion. Our results are in accordance with the findings of a previous study that reported that CovR deletion led to reduced virulence of Group B *Streptococcus* [27]. However, our study results are not in agreement with the assumption that CovR deletion leads to an increased virulence of *S. suis* 2 05ZYH33, Group A *Streptococcus* and *Streptococcus mutans* [25, 38, 39]. These results suggest that *S. suis* 2 can evolve different regulative mechanisms according to different living environments, and CovR plays a positive role in the pathogenesis of *S. suis* 2 SC19 isolated from a pig.

Although the above results indicate that CovR plays a positive regulative role in the pathogenesis of *S. suis* 2 SC19 stain in vivo and in vitro, the regulative mechanism of CovR in SC19 is unclear. Previous studies have revealed the pathogenic mechanism of bacteria escaping the host immune system by CovR regulation of a large array of virulence-associated genes [40]. However, meeting its own nutrient needs is another important survival problem of *S. suis* 2 after entering a new environment. We speculate that the regulation of metabolic pathways by regulative systems may play an important role in the use of nutrients in new environments. As expected, the KEGG pathway enrichment results showed that metabolic pathways, including fructose and mannose metabolism, glycerlipid metabolism, amino sugar and nucleotide sugar metabolism, ABC transporters and phosphotransferase system, were significantly down-regulated due to the deletion of CovR (Fig. 7B and Table S 4). Our results are also consistent with those of previous study which reported that the pathogenicity of *Streptococcus mutans* was attributed not only to the expression of virulence factors but also to its ability to respond and adapt rapidly to the ever-changing conditions of the oral cavity, including the availability of nutrients [33]. For example, *Streptococcus mutans* can metabolise the carbohydrates in the diet of the host to produce glucan, an extracellular sticky polysaccharide, which is necessary for anchoring to the tooth surface and the formation of dental plaque [41]. In addition, except for ABC transports, our results are different from micro array data suggesting that CovR deletion significantly changes the enzymes, transcriptional regulators, virulence-related factors and other proteins in *S. suis* 2 05HYH33 [25]. These results indicate that CovR plays an important role in bacterial rapid responses and adaptation to the ever-changing conditions, and CovR may drive the survivability and pathogenicity of *S. suis* 2 from pigs by regulating metabolic pathways.

In conclusion, our results demonstrate that CovR plays a positive regulative function in the pathogenicity of *S. suis* 2 SC19 strain isolated from a pig. Further studies suggest that CovR may regulate metabolic pathways, the ABC transporter pathway and the phosphotransferase system to allow *S. suis* 2 SC19 to rapidly adapt to the ever-changing environments. This study also suggests that CovR displays different regulative functions in *S. suis* 2 isolated from humans and pigs, implying that we should use different strategies when treating humans and pigs infected with *S. suis* 2. In our next study, the up- or down-regulated virulence genes will be screened based on transcriptome data.

### Supplementary Information

**Additional file 1: Supplementary table S1.** Sequences of the primersused for qRT-PCR.

**Additional file 2: Supplementary table S2.** 114 genes significantly down-regulated.

**Additional file 3: Supplementary table S3.** 117 genes significantly up-regulated.

**Additional file 4: Supplementary table S4.** qRT-PCR validation of genes related to the pathways of fructose and mannose metabolism, glycerolipid metabolism, ABC transporters, amino sugar and nucleotide sugar metabolism and phosphotransferase system (PTS)

**Additional file 5: Supplementary table S5.** The 128 areas (Identity < 85%) and 9 areas (Identity < 80%) between the genome of *S. suis* 2 SC19 and 05ZYH33 strains

**Additional file 6: Figure S1.** The identification of the knockout mutant Δ*covR* and complementation strain** C**Δ***covR. ***PCR confirmation of the mutant and CΔ*covR*. The primer pairs (the forward primer 5′-TCAATCGCGCATGGC-3′ and the reverse primer 5′-ATGGCTAAGAAAATTTTGATTG-3′ were used in the PCR, the 500 bp DNA fragment was amplified by using the primer pairs. Lane M indicated the DL5000 DNA Marker, lane 1 indicated the negative control without template, templates were genomic DNA from Δ*covR* (lane 2), CΔ*covR* (lane 3), and *S.suis* 2 SC19 (lane 4).** Figure S2 **The number of genes expressed in SC19 and Δ*covR*. **A **The Venn diagram showed 1823 genes are expressed inSC19 (Light red) and Δ*covR *(watchet). **B** Principal component analysis (PCA) of RNA-Seq data. The percentages on each axis represent the percentages of variation explained by the principal components. Points that are closer together are more similar in gene expression patterns. **Figure S3 **Differentially expressed genes of Δ*covR* compared to SC19. a Volcano plot showing the fold change (log2 ratio) in the expression of differentially expressed genes in Δ*covR* vs. SC19 (X-axis) plotted against the -log_10_ adjusted p-value (Y-axis). Each red square, gray dot and blue triangle on the plot represents the mean value (from three independent cultures) of one gene. Red square: Significantly up-regulated genes. Blue triangle: Significantly down-regulated genes. Gray dot: no significant difference genes. **Figure S4 **The GO andKEGG analysis of 114 down-regulated Genes.** A G**O annotations analysis. **B **KEGG analysis. **Figure S5** The GO annotations analysis of 117 up-regulated Genes. **Figure S6 **The collinearity analysis of *S. suis* 2 SC19 and 05ZYH33 strains. The genomes of *S. suis* 2 SC19 and 05ZYH33 strains are similar, however, the location and copy numbers of many gene elements in the genome were changed between *S. suis* 2 SC19 and 05ZYH33 strains

## Data Availability

The datasets generated and/or analysed during the current study are available in the [NCBI] repository, [https://www.ncbi.nlm.nih.gov/Traces/study/?acc=SRP397153&o=acc_s%3Aa. Accession: PRJNA879942. BioSamples: SAMN30820557/ SAMN30820556/ SAMN30820555/ SAMN30820554/ SAMN30820553/ SAMN30820552]”.
