# Supplementary material for: Orphan response regulator CovR plays positive regulative functions in the survivability and pathogenicity of Streptococcus suis serotype 2 isolated from a pig
Source: BMC Vet Res. 2023 Nov 22;19:243. doi: 10.1186/s12917-023-03808-9 (PMC10664645; doi:10.1186/s12917-023-03808-9)
Supplement: Supplementary file 1 — Additional file 1: Supplementary table S1. Sequences of the primersused for qRT-PCR. [file 12917_2023_3808_MOESM1_ESM.docx]

Supplementary table S1 Sequences of the primers used for qRT-PCR

| Gene | Forward (5′-3′) | Reverse (5′-3′) |
| --- | --- | --- |
| B9H01_RS07520 | ACACGAGGCAATGTGGCTAT | ACAAGGCATCTGGGTCTAAC |
| B9H01_RS08575 | AGTTCCTGCGGCTCTTCT | GCACCACCGTTACCTGAT |
| B9H01_RS01065 | TATTGCCCGCTTTACACG | CACCGACCAAACCGCCTA |
| B9H01_RS06090 | TGCTATTGCTCCAGAACG | CATTTCCGCATAGACATTTT |
| B9H01_RS09200 | GACTATCGCCATACAGGTAA | AAGAAAGGAAATAAGGGAAC |
| B9H01_RS01845  B9H01_RS09195  B9H01_RS09190  B9H01_RS04035  B9H01_RS10010  B9H01_RS10265  B9H01_RS10260  B9H01_RS03575  B9H01_RS01850  B9H01_RS01165  B9H01_RS04040  B9H01_RS10025  B9H01_RS08580  B9H01_RS10000  B9H01_RS09285  B9H01_RS10255  B9H01_RS10005 | TCTTTCCAGCGGCTATTA  AAATCTGCTTGCCACTAAC  AGCGACTACTGACGCAACCT  TTGTCTTTGCTGGGTCTG  CAGCGTCAGCGGGTATTT  GTGGTGGTATCCCAATGAA  ATGCTGATTCGTGGTTCTT  TGTGACGGATTATTCAAACG  CCAACTCTGACCTCCCT  GTATTTATGGGCGGTGGTG  GAATGATGGCAGTCGATAT  TGCTCTGCTATTGTCCCTAA  AAGAAGAGCGTGCTAATG  TCTGGCTTACGAACAAGG  GGAATGAATGCGGCGAA  TTGCTAATGAAGGTGCTA  AGGAGCAGTGTTGTCAGG | TGATGACAAGCCTGAACC  ACGACCACAATAGCCATAC  CTCCCTCTGGACCAAATACC  TAGCGTAGGTTTCAATGTCT  CATCCATCGGACCAGAGT  AAGGTTTGAAGTCCGACAG  CTTGGTGGCAGGTGTTTGA  ATACCTGGCTCAAATGCTA  GCCTTAATCTGAACGCTA  CATCGTCCAAGGGAGAACA  CAGTGATGAATGAAAGACCC  TCAAAGTGCAAACCGTGAT  AATGATGTTCCAGCCAAG  ACAAGAAGGCTGACAACG  AAAGCAACCAGACCACCC  TTTGCCGTTTACAAGAGT  ATGCCAAAGCCAATAGAG |
