# Supplementary material for: Orphan response regulator CovR plays positive regulative functions in the survivability and pathogenicity of Streptococcus suis serotype 2 isolated from a pig
Source: BMC Vet Res. 2023 Nov 22;19:243. doi: 10.1186/s12917-023-03808-9 (PMC10664645; doi:10.1186/s12917-023-03808-9)
Supplement: Supplementary file 2 — Additional file 2: Supplementary table S2. 114 genes significantly down-regulated. [file 12917_2023_3808_MOESM2_ESM.docx]

Supplementary table S2 114 genes significantly down-regulated

| Gene_id | Gene name | Gene description | FC(covR/SC19) | Log2FC(covR/SC19) | Pvalue | Padjust | Significant | Regulate |
| --- | --- | --- | --- | --- | --- | --- | --- | --- |
| B9H01_RS08355 | B9H01_RS08355 | winged helix-turn-helix domain-containing protein | 0 | -12.4586 | 5.81E-26 | 1.08E-23 | yes | down |
| B9H01_RS09935 | B9H01_RS09935 | amino acid ABC transporter permease | 0.607 | -0.71978 | 0.004469 | 0.036404 | yes | down |
| B9H01_RS07505 | B9H01_RS07505 | carbohydrate ABC transporter permease | 0.619 | -0.69261 | 2.69E-06 | 6.80E-05 | yes | down |
| B9H01_RS09415 | B9H01_RS09415 | PTS-dependent dihydroxyacetone kinase phosphotransferase subunit DhaM | 0.633 | -0.65927 | 7.36E-15 | 8.78E-13 | yes | down |
| B9H01_RS07540 | B9H01_RS07540 | hypothetical protein | 0.635 | -0.65528 | 7.06E-05 | 0.001156 | yes | down |
| B9H01_RS09405 | dhaK | dihydroxyacetone kinase subunit DhaK | 0.642 | -0.64045 | 4.02E-19 | 6.71E-17 | yes | down |
| B9H01_RS06175 | B9H01_RS06175 | DUF2304 domain-containing protein | 0.647 | -0.62743 | 0.000766 | 0.009008 | yes | down |
| B9H01_RS09410 | dhaL | dihydroxyacetone kinase subunit L | 0.663 | -0.59395 | 1.22E-17 | 1.85E-15 | yes | down |
| B9H01_RS09420 | B9H01_RS09420 | aquaporin family protein | 0.665 | -0.58815 | 1.31E-16 | 1.82E-14 | yes | down |
| B9H01_RS07520 | B9H01_RS07520 | alpha-galactosidase | 0.666 | -0.58558 | 0.000335 | 0.004543 | yes | down |
| B9H01_RS07585 | B9H01_RS07585 | autorepressor SdpR family transcription factor | 0.675 | -0.5666 | 1.02E-07 | 4.05E-06 | yes | down |
| B9H01_RS01560 | dnaK | molecular chaperone DnaK | 0.683 | -0.55096 | 1.48E-14 | 1.65E-12 | yes | down |
| B9H01_RS09200 | B9H01_RS09200 | sugar ABC transporter permease | 0.683 | -0.55054 | 3.21E-08 | 1.49E-06 | yes | down |
| B9H01_RS10500 | B9H01_RS10500 | YfhO family protein | 0.684 | -0.54799 | 3.34E-16 | 4.29E-14 | yes | down |
| B9H01_RS08010 | B9H01_RS08010 | isoprenylcysteine carboxyl methyltransferase family protein | 0.686 | -0.54285 | 0.002629 | 0.024058 | yes | down |
| B9H01_RS09195 | B9H01_RS09195 | carbohydrate ABC transporter permease | 0.695 | -0.52549 | 4.60E-07 | 1.45E-05 | yes | down |
| B9H01_RS02160 | cysK | cysteine synthase A | 0.695 | -0.52494 | 4.95E-05 | 0.000864 | yes | down |
| B9H01_RS09940 | B9H01_RS09940 | amino acid ABC transporter substrate-binding protein | 0.698 | -0.51921 | 1.50E-06 | 4.04E-05 | yes | down |
| B9H01_RS01550 | hrcA | heat-inducible transcriptional repressor HrcA | 0.7 | -0.51442 | 2.11E-12 | 1.76E-10 | yes | down |
| B9H01_RS07580 | B9H01_RS07580 | DUF1648 domain-containing protein | 0.703 | -0.50837 | 5.27E-06 | 0.000117 | yes | down |
| B9H01_RS02560 | B9H01_RS02560 | U32 family peptidase | 0.71 | -0.49509 | 1.22E-09 | 6.59E-08 | yes | down |
| B9H01_RS01555 | grpE | nucleotide exchange factor GrpE | 0.711 | -0.49219 | 1.86E-10 | 1.03E-08 | yes | down |
| B9H01_RS06440 | B9H01_RS06440 | DUF3397 domain-containing protein | 0.717 | -0.47952 | 0.005528 | 0.041721 | yes | down |
| B9H01_RS01065 | B9H01_RS01065 | PTS ascorbate transporter subunit IIC | 0.721 | -0.47281 | 0.000276 | 0.003907 | yes | down |
| B9H01_RS00765 | B9H01_RS00765 | HIT family protein | 0.723 | -0.46723 | 0.000112 | 0.001716 | yes | down |
| B9H01_RS03200 | dltA | D-alanine--poly(phosphoribitol) ligase subunit DltA | 0.726 | -0.4626 | 4.96E-05 | 0.000864 | yes | down |
| B9H01_RS07500 | gtfA | sucrose phosphorylase | 0.73 | -0.45406 | 0.001251 | 0.013393 | yes | down |
| B9H01_RS08415 | B9H01_RS08415 | DUF3278 domain-containing protein | 0.733 | -0.44749 | 8.15E-07 | 2.39E-05 | yes | down |
| B9H01_RS09220 | B9H01_RS09220 | glycoside hydrolase family 125 protein | 0.738 | -0.43874 | 3.70E-07 | 1.25E-05 | yes | down |
| B9H01_RS08430 | B9H01_RS08430 | hypothetical protein | 0.745 | -0.42433 | 8.16E-07 | 2.39E-05 | yes | down |
| B9H01_RS09190 | B9H01_RS09190 | ABC transporter substrate-binding protein | 0.747 | -0.42035 | 1.61E-06 | 4.27E-05 | yes | down |
| B9H01_RS08425 | B9H01_RS08425 | DUF3278 domain-containing protein | 0.748 | -0.41973 | 5.15E-07 | 1.59E-05 | yes | down |
| B9H01_RS01165 | B9H01_RS01165 | PTS sugar transporter subunit IIC | 0.75 | -0.41563 | 5.15E-08 | 2.21E-06 | yes | down |
| B9H01_RS10070 | sntA | heme-binding protein SntA | 0.75 | -0.4156 | 2.25E-07 | 8.56E-06 | yes | down |
| B9H01_RS01055 | B9H01_RS01055 | BglG family transcription antiterminator | 0.75 | -0.41496 | 0.000185 | 0.002688 | yes | down |
| B9H01_RS08410 | B9H01_RS08410 | helix-turn-helix transcriptional regulator | 0.751 | -0.4131 | 0.000683 | 0.008268 | yes | down |
| B9H01_RS09095 | B9H01_RS09095 | DUF6176 family protein | 0.754 | -0.40776 | 0.00073 | 0.008707 | yes | down |
| B9H01_RS01845 | B9H01_RS01845 | galactokinase | 0.764 | -0.38764 | 9.39E-09 | 4.61E-07 | yes | down |
| B9H01_RS07465 | B9H01_RS07465 | branched-chain amino acid ABC transporter permease | 0.764 | -0.38855 | 0.002623 | 0.024058 | yes | down |
| B9H01_RS10025 | B9H01_RS10025 | zinc ABC transporter substrate-binding protein | 0.766 | -0.3842 | 1.76E-08 | 8.40E-07 | yes | down |
| B9H01_RS01045 | B9H01_RS01045 | glycoside hydrolase family 1 protein | 0.766 | -0.38506 | 0.00029 | 0.004067 | yes | down |
| B9H01_RS05395 | B9H01_RS05395 | Spx/MgsR family RNA polymerase-binding regulatory protein | 0.774 | -0.36968 | 0.0001 | 0.001567 | yes | down |
| B9H01_RS09210 | B9H01_RS09210 | ROK family protein | 0.776 | -0.36581 | 6.46E-05 | 0.001068 | yes | down |
| B9H01_RS01840 | B9H01_RS01840 | LacI family DNA-binding transcriptional regulator | 0.778 | -0.36282 | 5.17E-08 | 2.21E-06 | yes | down |
| B9H01_RS03135 | argF | ornithine carbamoyltransferase | 0.778 | -0.36259 | 6.34E-08 | 2.65E-06 | yes | down |
| B9H01_RS09230 | B9H01_RS09230 | bacterial Ig-like domain-containing protein | 0.778 | -0.36157 | 4.28E-05 | 0.000786 | yes | down |
| B9H01_RS01565 | dnaJ | molecular chaperone DnaJ | 0.782 | -0.35424 | 1.39E-06 | 3.87E-05 | yes | down |
| B9H01_RS05300 | coaA | type I pantothenate kinase | 0.786 | -0.34822 | 4.33E-06 | 0.000101 | yes | down |
| B9H01_RS10240 | B9H01_RS10240 | hypothetical protein | 0.791 | -0.33901 | 0.001189 | 0.013001 | yes | down |
| B9H01_RS10495 | B9H01_RS10495 | ATP-binding cassette domain-containing protein | 0.792 | -0.33705 | 3.16E-07 | 1.15E-05 | yes | down |
| B9H01_RS08575 | B9H01_RS08575 | PTS mannose/fructose/sorbose transporter subunit IIC | 0.792 | -0.33574 | 0.000113 | 0.001716 | yes | down |
| B9H01_RS07915 | B9H01_RS07915 | phosphoglycerate mutase | 0.796 | -0.3299 | 0.005737 | 0.042775 | yes | down |
| B9H01_RS01170 | B9H01_RS01170 | DUF4838 domain-containing protein | 0.797 | -0.32766 | 7.52E-06 | 0.000163 | yes | down |
| B9H01_RS10285 | nrdI | class Ib ribonucleoside-diphosphate reductase assembly flavoprotein NrdI | 0.797 | -0.32815 | 0.000493 | 0.006377 | yes | down |
| B9H01_RS10010 | B9H01_RS10010 | metal ABC transporter ATP-binding protein | 0.799 | -0.32371 | 3.48E-06 | 8.42E-05 | yes | down |
| B9H01_RS09455 | gshAB | bifunctional glutamate--cysteine ligase GshA/glutathione synthetase GshB | 0.8 | -0.32108 | 7.46E-08 | 3.04E-06 | yes | down |
| B9H01_RS01410 | B9H01_RS01410 | aquaporin family protein | 0.8 | -0.32114 | 0.004126 | 0.034114 | yes | down |
| B9H01_RS09215 | B9H01_RS09215 | alpha-mannosidase | 0.801 | -0.32021 | 2.61E-05 | 0.000496 | yes | down |
| B9H01_RS00200 | B9H01_RS00200 | CHAP domain-containing protein | 0.801 | -0.31993 | 0.000149 | 0.002244 | yes | down |
| B9H01_RS08435 | B9H01_RS08435 | hypothetical protein | 0.801 | -0.31931 | 0.000663 | 0.008082 | yes | down |
| B9H01_RS09285 | B9H01_RS09285 | PTS transporter subunit EIIC | 0.801 | -0.32089 | 0.001964 | 0.019404 | yes | down |
| B9H01_RS10005 | B9H01_RS10005 | metal ABC transporter permease | 0.802 | -0.31787 | 3.73E-07 | 1.25E-05 | yes | down |
| B9H01_RS10305 | B9H01_RS10305 | aldo/keto reductase | 0.804 | -0.31431 | 0.002491 | 0.023237 | yes | down |
| B9H01_RS08505 | glyS | glycine--tRNA ligase subunit beta | 0.805 | -0.31361 | 1.58E-05 | 0.000321 | yes | down |
| B9H01_RS10195 | nrdD | anaerobic ribonucleoside-triphosphate reductase | 0.807 | -0.31018 | 4.41E-06 | 0.000101 | yes | down |
| B9H01_RS03045 | B9H01_RS03045 | NAD(P)H-dependent oxidoreductase | 0.813 | -0.29817 | 0.005546 | 0.041721 | yes | down |
| B9H01_RS07550 | B9H01_RS07550 | hypothetical protein | 0.814 | -0.29626 | 0.000164 | 0.00245 | yes | down |
| B9H01_RS03585 | B9H01_RS03585 | aquaporin family protein | 0.816 | -0.29348 | 3.43E-06 | 8.42E-05 | yes | down |
| B9H01_RS03130 | B9H01_RS03130 | GNAT family N-acetyltransferase | 0.817 | -0.29075 | 1.13E-05 | 0.000235 | yes | down |
| B9H01_RS06090 | nagA | N-acetylglucosamine-6-phosphate deacetylase | 0.817 | -0.2921 | 0.003853 | 0.032994 | yes | down |
| B9H01_RS04040 | B9H01_RS04040 | fructose-specific PTS transporter subunit EIIC | 0.82 | -0.28687 | 4.16E-07 | 1.34E-05 | yes | down |
| B9H01_RS01850 | galT | UDP-glucose--hexose-1-phosphate uridylyltransferase | 0.82 | -0.28679 | 1.06E-06 | 3.01E-05 | yes | down |
| B9H01_RS09205 | B9H01_RS09205 | beta-N-acetylhexosaminidase | 0.823 | -0.28064 | 0.000491 | 0.006377 | yes | down |
| B9H01_RS08440 | B9H01_RS08440 | NusG domain II-containing protein | 0.828 | -0.27202 | 0.0001 | 0.001567 | yes | down |
| B9H01_RS03125 | arcA | arginine deiminase | 0.829 | -0.27105 | 0.000219 | 0.003158 | yes | down |
| B9H01_RS10255 | B9H01_RS10255 | extracellular solute-binding protein | 0.83 | -0.26872 | 0.000497 | 0.006389 | yes | down |
| B9H01_RS03455 | B9H01_RS03455 | FAD-containing oxidoreductase | 0.831 | -0.26771 | 0.00236 | 0.022517 | yes | down |
| B9H01_RS08580 | B9H01_RS08580 | PTS system mannose/fructose/sorbose family transporter subunit IID | 0.832 | -0.26462 | 0.000166 | 0.002458 | yes | down |
| B9H01_RS03580 | glpO | type 1 glycerol-3-phosphate oxidase | 0.833 | -0.26443 | 1.60E-05 | 0.000321 | yes | down |
| B9H01_RS10260 | B9H01_RS10260 | sugar ABC transporter permease | 0.834 | -0.26129 | 0.000326 | 0.004463 | yes | down |
| B9H01_RS05295 | B9H01_RS05295 | class I SAM-dependent methyltransferase | 0.837 | -0.25687 | 5.16E-05 | 0.000879 | yes | down |
| B9H01_RS08510 | glyQ | glycine--tRNA ligase subunit alpha | 0.838 | -0.25549 | 0.002546 | 0.023623 | yes | down |
| B9H01_RS05965 | B9H01_RS05965 | DUF3267 domain-containing protein | 0.839 | -0.2537 | 0.000467 | 0.006134 | yes | down |
| B9H01_RS03140 | arcC | carbamate kinase | 0.84 | -0.25186 | 2.78E-05 | 0.000521 | yes | down |
| B9H01_RS03150 | B9H01_RS03150 | dipeptidase | 0.841 | -0.24977 | 0.000109 | 0.001687 | yes | down |
| novel0002 | - | transposase, partial [Streptococcus suis] | 0.841 | -0.24964 | 0.001351 | 0.014096 | yes | down |
| B9H01_RS03475 | B9H01_RS03475 | hypothetical protein | 0.841 | -0.24899 | 0.002452 | 0.023111 | yes | down |
| B9H01_RS10265 | B9H01_RS10265 | sugar ABC transporter permease | 0.843 | -0.24566 | 0.004123 | 0.034114 | yes | down |
| B9H01_RS09530 | B9H01_RS09530 | S8 family serine peptidase | 0.849 | -0.23692 | 0.00126 | 0.013401 | yes | down |
| B9H01_RS03575 | glpK | glycerol kinase GlpK | 0.85 | -0.23438 | 0.002313 | 0.022202 | yes | down |
| B9H01_RS01095 | B9H01_RS01095 | YSIRK signal domain/LPXTG anchor domain surface protein | 0.853 | -0.2294 | 0.004616 | 0.037419 | yes | down |
| B9H01_RS07645 | glmU | bifunctional UDP-N-acetylglucosamine diphosphorylase/glucosamine-1-phosphate N-acetyltransferase GlmU | 0.854 | -0.22804 | 0.000877 | 0.01017 | yes | down |
| B9H01_RS05955 | B9H01_RS05955 | DUF3169 family protein | 0.855 | -0.22559 | 0.003064 | 0.027512 | yes | down |
| B9H01_RS10270 | B9H01_RS10270 | integral membrane protein | 0.856 | -0.22381 | 0.005388 | 0.041277 | yes | down |
| B9H01_RS03725 | B9H01_RS03725 | MucBP domain-containing protein | 0.857 | -0.22271 | 0.000919 | 0.010509 | yes | down |
| B9H01_RS09225 | B9H01_RS09225 | GH92 family glycosyl hydrolase | 0.857 | -0.22298 | 0.003612 | 0.031258 | yes | down |
| novel0024 | - | transposase, partial [Streptococcus suis] | 0.858 | -0.22153 | 0.004902 | 0.039546 | yes | down |
| B9H01_RS10155 | B9H01_RS10155 | replication-associated recombination protein A | 0.859 | -0.21853 | 0.003589 | 0.031214 | yes | down |
| B9H01_RS10185 | B9H01_RS10185 | 5'-nucleotidase C-terminal domain-containing protein | 0.86 | -0.21718 | 0.002758 | 0.025034 | yes | down |
| B9H01_RS10280 | pulA | type I pullulanase | 0.862 | -0.21357 | 0.000313 | 0.004316 | yes | down |
| B9H01_RS10000 | B9H01_RS10000 | metal ABC transporter permease | 0.864 | -0.2114 | 0.000341 | 0.004543 | yes | down |
| B9H01_RS01385 | B9H01_RS01385 | DUF1700 domain-containing protein | 0.866 | -0.20714 | 0.003056 | 0.027512 | yes | down |
| B9H01_RS10090 | B9H01_RS10090 | class C sortase | 0.869 | -0.20287 | 0.000889 | 0.010242 | yes | down |
| B9H01_RS10275 | B9H01_RS10275 | LacI family DNA-binding transcriptional regulator | 0.869 | -0.20293 | 0.001327 | 0.01394 | yes | down |
| B9H01_RS07615 | B9H01_RS07615 | hypothetical protein | 0.873 | -0.1967 | 0.002636 | 0.024058 | yes | down |
| B9H01_RS08385 | B9H01_RS08385 | hypothetical protein | 0.874 | -0.19383 | 0.005323 | 0.041155 | yes | down |
| B9H01_RS10080 | B9H01_RS10080 | class C sortase | 0.875 | -0.19281 | 0.00066 | 0.008082 | yes | down |
| B9H01_RS07760 | manA | mannose-6-phosphate isomerase%2C class I | 0.875 | -0.19298 | 0.005189 | 0.040684 | yes | down |
| B9H01_RS09460 | B9H01_RS09460 | DUF6359 domain-containing protein | 0.876 | -0.19116 | 0.005375 | 0.041277 | yes | down |
| B9H01_RS04035 | pfkB | 1-phosphofructokinase | 0.877 | -0.18854 | 0.001896 | 0.018845 | yes | down |
| B9H01_RS01160 | B9H01_RS01160 | ROK family protein | 0.885 | -0.17567 | 0.00338 | 0.029709 | yes | down |
| B9H01_RS01285 | B9H01_RS01285 | AMP-binding protein | 0.889 | -0.1704 | 0.002057 | 0.020091 | yes | down |
| B9H01_RS05275 | B9H01_RS05275 | BMP family protein | 0.904 | -0.1455 | 0.005526 | 0.041721 | yes | down |
| B9H01_RS05280 | B9H01_RS05280 | cytidine deaminase | 0.904 | -0.14619 | 0.0063 | 0.045946 | yes | down |

Note: DESeq2 software was used for difference analysis.
