# Supplementary material for: Orphan response regulator CovR plays positive regulative functions in the survivability and pathogenicity of Streptococcus suis serotype 2 isolated from a pig
Source: BMC Vet Res. 2023 Nov 22;19:243. doi: 10.1186/s12917-023-03808-9 (PMC10664645; doi:10.1186/s12917-023-03808-9)
Supplement: Supplementary file 3 — Additional file 3: Supplementary table S3. 117 genes significantly up-regulated. [file 12917_2023_3808_MOESM3_ESM.docx]

Supplementary table S3 117 genes significantly up-regulated

| Gene_id | Gene name | Gene description | FC(covR/SC19) | Log2FC(covR/SC19) | Pvalue | Padjust | Significant | Regulate |
| --- | --- | --- | --- | --- | --- | --- | --- | --- |
| B9H01_RS01335 | gdhA | NADP-specific glutamate dehydrogenase | 29.241 | 4.869901 | 0 | 0 | yes | up |
| B9H01_RS01495 | B9H01_RS01495 | ABC transporter ATP-binding protein | 2.603 | 1.380375 | 4.52E-108 | 1.89E-105 | yes | up |
| B9H01_RS01490 | B9H01_RS01490 | energy-coupling factor transporter transmembrane protein EcfT | 2.536 | 1.342691 | 2.97E-41 | 7.09E-39 | yes | up |
| B9H01_RS01480 | B9H01_RS01480 | ABC transporter ATP-binding protein/permease | 2.439 | 1.28655 | 1.99E-121 | 1.66E-118 | yes | up |
| B9H01_RS01475 | B9H01_RS01475 | ABC transporter ATP-binding protein/permease | 2.415 | 1.272279 | 1.30E-110 | 7.25E-108 | yes | up |
| B9H01_RS01485 | B9H01_RS01485 | MptD family putative ECF transporter S component | 2.366 | 1.242253 | 4.73E-48 | 1.32E-45 | yes | up |
| B9H01_RS01500 | B9H01_RS01500 | MATE family efflux transporter | 2.353 | 1.23467 | 4.94E-82 | 1.65E-79 | yes | up |
| B9H01_RS03780 | B9H01_RS03780 | branched-chain amino acid aminotransferase | 2.194 | 1.133677 | 2.03E-14 | 2.12E-12 | yes | up |
| B9H01_RS06580 | B9H01_RS06580 | amino acid ABC transporter permease | 2.095 | 1.06691 | 1.44E-11 | 1.09E-09 | yes | up |
| B9H01_RS04995 | B9H01_RS04995 | transporter substrate-binding domain-containing protein | 2.025 | 1.017579 | 0.001155 | 0.012769 | yes | up |
| B9H01_RS06575 | B9H01_RS06575 | transporter substrate-binding domain-containing protein | 2.001 | 1.000978 | 6.75E-11 | 4.17E-09 | yes | up |
| B9H01_RS09035 | B9H01_RS09035 | ABC transporter substrate-binding protein/permease | 1.924 | 0.944099 | 4.80E-29 | 1.00E-26 | yes | up |
| B9H01_RS06570 | B9H01_RS06570 | amino acid ABC transporter ATP-binding protein | 1.919 | 0.940389 | 2.77E-06 | 6.89E-05 | yes | up |
| B9H01_RS06585 | B9H01_RS06585 | amino acid ABC transporter permease | 1.781 | 0.832447 | 4.10E-06 | 9.77E-05 | yes | up |
| B9H01_RS09040 | B9H01_RS09040 | amino acid ABC transporter ATP-binding protein | 1.715 | 0.777908 | 6.16E-09 | 3.12E-07 | yes | up |
| B9H01_RS03255 | B9H01_RS03255 | ABC transporter ATP-binding protein | 1.654 | 0.726117 | 1.61E-12 | 1.42E-10 | yes | up |
| B9H01_RS01965 | B9H01_RS01965 | ATP-dependent Clp protease ATP-binding subunit | 1.631 | 0.705954 | 0.001763 | 0.018054 | yes | up |
| B9H01_RS03245 | B9H01_RS03245 | iron ABC transporter permease | 1.617 | 0.693281 | 6.04E-11 | 4.04E-09 | yes | up |
| B9H01_RS03240 | B9H01_RS03240 | iron ABC transporter permease | 1.616 | 0.692643 | 5.42E-12 | 4.31E-10 | yes | up |
| B9H01_RS03480 | B9H01_RS03480 | DUF6261 family protein | 1.554 | 0.636409 | 1.78E-06 | 4.65E-05 | yes | up |
| B9H01_RS06805 | feoB | ferrous iron transport protein B | 1.516 | 0.600469 | 6.75E-11 | 4.17E-09 | yes | up |
| B9H01_RS00690 | B9H01_RS00690 | replication initiation factor domain-containing protein | 1.51 | 0.594617 | 3.98E-07 | 1.30E-05 | yes | up |
| B9H01_RS03250 | B9H01_RS03250 | ABC transporter substrate-binding protein | 1.505 | 0.589893 | 1.42E-09 | 7.41E-08 | yes | up |
| B9H01_RS09015 | sufC | Fe-S cluster assembly ATPase SufC | 1.501 | 0.585648 | 2.17E-13 | 2.13E-11 | yes | up |
| B9H01_RS02495 | B9H01_RS02495 | amino acid ABC transporter permease | 1.488 | 0.573369 | 0.004971 | 0.039688 | yes | up |
| B9H01_RS09010 | sufD | Fe-S cluster assembly protein SufD | 1.464 | 0.549657 | 3.68E-11 | 2.56E-09 | yes | up |
| B9H01_RS00680 | B9H01_RS00680 | site-specific integrase | 1.445 | 0.531296 | 3.37E-08 | 1.52E-06 | yes | up |
| B9H01_RS09005 | B9H01_RS09005 | cysteine desulfurase | 1.441 | 0.527251 | 1.33E-10 | 7.65E-09 | yes | up |
| B9H01_RS08810 | B9H01_RS08810 | single-stranded DNA-binding protein | 1.437 | 0.522727 | 0.000966 | 0.010897 | yes | up |
| B9H01_RS09650 | B9H01_RS09650 | type II toxin-antitoxin system Phd/YefM family antitoxin | 1.436 | 0.522516 | 0.000343 | 0.004543 | yes | up |
| B9H01_RS09000 | B9H01_RS09000 | SUF system NifU family Fe-S cluster assembly protein | 1.434 | 0.520014 | 1.30E-10 | 7.65E-09 | yes | up |
| B9H01_RS08995 | sufB | Fe-S cluster assembly protein SufB | 1.424 | 0.509693 | 2.18E-11 | 1.58E-09 | yes | up |
| B9H01_RS00695 | B9H01_RS00695 | hypothetical protein | 1.423 | 0.509326 | 0.000553 | 0.006945 | yes | up |
| B9H01_RS03360 | B9H01_RS03360 | amino acid permease | 1.419 | 0.50512 | 0.002051 | 0.020091 | yes | up |
| B9H01_RS01670 | B9H01_RS01670 | Rrf2 family transcriptional regulator | 1.403 | 0.488413 | 0.000716 | 0.008598 | yes | up |
| B9H01_RS02775 | B9H01_RS02775 | amino acid ABC transporter permease | 1.392 | 0.477431 | 1.06E-06 | 3.01E-05 | yes | up |
| B9H01_RS00445 | rplC | 50S ribosomal protein L3 | 1.385 | 0.469487 | 2.47E-07 | 9.16E-06 | yes | up |
| B9H01_RS02785 | B9H01_RS02785 | amino acid ABC transporter substrate-binding protein | 1.369 | 0.453394 | 6.24E-06 | 0.000137 | yes | up |
| B9H01_RS02780 | B9H01_RS02780 | amino acid ABC transporter ATP-binding protein | 1.366 | 0.450342 | 1.09E-05 | 0.00023 | yes | up |
| B9H01_RS00715 | B9H01_RS00715 | hypothetical protein | 1.361 | 0.444597 | 0.002155 | 0.020923 | yes | up |
| B9H01_RS06680 | B9H01_RS06680 | heavy metal translocating P-type ATPase | 1.351 | 0.433722 | 2.33E-13 | 2.17E-11 | yes | up |
| B9H01_RS00440 | rpsJ | 30S ribosomal protein S10 | 1.336 | 0.41739 | 2.09E-05 | 0.000406 | yes | up |
| B9H01_RS08650 | B9H01_RS08650 | acetyl-CoA carboxylase biotin carboxylase subunit | 1.327 | 0.408596 | 9.88E-05 | 0.001567 | yes | up |
| B9H01_RS03595 | B9H01_RS03595 | hypothetical protein | 1.326 | 0.407314 | 0.002233 | 0.021556 | yes | up |
| B9H01_RS09635 | rsmA | 16S rRNA (adenine(1518)-N(6)/adenine(1519)-N(6))-dimethyltransferase RsmA | 1.321 | 0.401463 | 2.12E-05 | 0.000407 | yes | up |
| B9H01_RS00450 | rplD | 50S ribosomal protein L4 | 1.308 | 0.387587 | 4.43E-06 | 0.000101 | yes | up |
| B9H01_RS05035 | B9H01_RS05035 | ABC transporter substrate-binding protein/permease | 1.304 | 0.383223 | 4.52E-06 | 0.000102 | yes | up |
| B9H01_RS03220 | B9H01_RS03220 | low temperature requirement protein A | 1.297 | 0.37505 | 0.000179 | 0.002621 | yes | up |
| B9H01_RS00700 | B9H01_RS00700 | cell division protein FtsK | 1.297 | 0.375396 | 0.005864 | 0.043334 | yes | up |
| B9H01_RS05610 | B9H01_RS05610 | HAD family hydrolase | 1.293 | 0.370218 | 3.39E-07 | 1.21E-05 | yes | up |
| B9H01_RS01660 | B9H01_RS01660 | SDR family NAD(P)-dependent oxidoreductase | 1.293 | 0.370762 | 0.001201 | 0.013027 | yes | up |
| B9H01_RS08640 | B9H01_RS08640 | acetyl-CoA carboxylase carboxyl transferase subunit alpha | 1.293 | 0.370265 | 0.004391 | 0.035945 | yes | up |
| B9H01_RS08660 | accB | acetyl-CoA carboxylase biotin carboxyl carrier protein | 1.285 | 0.36164 | 0.005716 | 0.042775 | yes | up |
| B9H01_RS00455 | B9H01_RS00455 | 50S ribosomal protein L23 | 1.281 | 0.357129 | 7.93E-05 | 0.001273 | yes | up |
| B9H01_RS05515 | B9H01_RS05515 | MarR family transcriptional regulator | 1.279 | 0.354932 | 8.05E-06 | 0.000172 | yes | up |
| B9H01_RS05625 | uxaC | glucuronate isomerase | 1.273 | 0.347729 | 1.54E-05 | 0.000318 | yes | up |
| B9H01_RS03225 | B9H01_RS03225 | glutathione S-transferase family protein | 1.267 | 0.341026 | 0.003917 | 0.033204 | yes | up |
| B9H01_RS04325 | xerS | tyrosine recombinase XerS | 1.264 | 0.337973 | 0.003301 | 0.029169 | yes | up |
| B9H01_RS05740 | B9H01_RS05740 | DNA alkylation repair protein | 1.263 | 0.336798 | 4.38E-05 | 0.000795 | yes | up |
| B9H01_RS05745 | B9H01_RS05745 | GNAT family N-acetyltransferase | 1.259 | 0.331936 | 0.001212 | 0.013059 | yes | up |
| B9H01_RS00560 | B9H01_RS00560 | adenylate kinase | 1.258 | 0.330711 | 0.001524 | 0.015707 | yes | up |
| B9H01_RS09800 | B9H01_RS09800 | MarR family transcriptional regulator | 1.256 | 0.328352 | 0.000574 | 0.007157 | yes | up |
| B9H01_RS05600 | B9H01_RS05600 | glycoside hydrolase family 3 protein | 1.255 | 0.328181 | 1.61E-05 | 0.000321 | yes | up |
| B9H01_RS05245 | gyrA | DNA gyrase subunit A | 1.254 | 0.32667 | 5.47E-05 | 0.000923 | yes | up |
| B9H01_RS01685 | B9H01_RS01685 | alpha/beta hydrolase | 1.253 | 0.325656 | 0.006201 | 0.045621 | yes | up |
| B9H01_RS01665 | B9H01_RS01665 | NADH:flavin oxidoreductase | 1.246 | 0.317808 | 0.000543 | 0.006864 | yes | up |
| B9H01_RS05620 | B9H01_RS05620 | mannonate dehydratase | 1.24 | 0.310844 | 1.68E-05 | 0.00033 | yes | up |
| B9H01_RS05605 | B9H01_RS05605 | beta-hexosamidase | 1.24 | 0.310102 | 4.97E-05 | 0.000864 | yes | up |
| B9H01_RS05240 | B9H01_RS05240 | class A sortase | 1.237 | 0.30722 | 0.000796 | 0.009301 | yes | up |
| B9H01_RS09795 | B9H01_RS09795 | ABC transporter ATP-binding protein/permease | 1.235 | 0.304117 | 6.46E-05 | 0.001068 | yes | up |
| B9H01_RS04200 | B9H01_RS04200 | hypothetical protein | 1.233 | 0.302268 | 0.00074 | 0.008763 | yes | up |
| B9H01_RS05615 | B9H01_RS05615 | SDR family oxidoreductase | 1.229 | 0.297081 | 4.09E-05 | 0.000758 | yes | up |
| B9H01_RS04305 | B9H01_RS04305 | DUF3307 domain-containing protein | 1.227 | 0.295311 | 1.46E-06 | 4.00E-05 | yes | up |
| B9H01_RS08150 | B9H01_RS08150 | DNA starvation/stationary phase protection protein | 1.226 | 0.29405 | 0.000343 | 0.004543 | yes | up |
| B9H01_RS01645 | B9H01_RS01645 | Rrf2 family transcriptional regulator | 1.226 | 0.293485 | 0.005304 | 0.041155 | yes | up |
| B9H01_RS07920 | B9H01_RS07920 | acetyl-CoA C-acetyltransferase | 1.225 | 0.292196 | 0.000261 | 0.003731 | yes | up |
| B9H01_RS05040 | B9H01_RS05040 | amino acid ABC transporter ATP-binding protein | 1.224 | 0.291672 | 7.90E-05 | 0.001273 | yes | up |
| B9H01_RS03510 | B9H01_RS03510 | DeoR/GlpR family DNA-binding transcription regulator | 1.224 | 0.291699 | 0.001366 | 0.014167 | yes | up |
| B9H01_RS05630 | eda | bifunctional 4-hydroxy-2-oxoglutarate aldolase/2-dehydro-3-deoxy-phosphogluconate aldolase | 1.224 | 0.291077 | 0.001773 | 0.018054 | yes | up |
| B9H01_RS05735 | zwf | glucose-6-phosphate dehydrogenase | 1.221 | 0.287511 | 1.64E-07 | 6.38E-06 | yes | up |
| B9H01_RS01355 | B9H01_RS01355 | ABC-2 family transporter protein | 1.216 | 0.281599 | 6.48E-07 | 1.97E-05 | yes | up |
| B9H01_RS03345 | B9H01_RS03345 | cation transporter | 1.215 | 0.281543 | 3.62E-07 | 1.25E-05 | yes | up |
| B9H01_RS09790 | B9H01_RS09790 | ABC transporter ATP-binding protein/permease | 1.212 | 0.277503 | 4.74E-05 | 0.000852 | yes | up |
| B9H01_RS01365 | B9H01_RS01365 | ATP-binding cassette domain-containing protein | 1.211 | 0.276644 | 2.67E-06 | 6.80E-05 | yes | up |
| B9H01_RS00470 | rplV | 50S ribosomal protein L22 | 1.211 | 0.276188 | 0.00031 | 0.004316 | yes | up |
| B9H01_RS00465 | rpsS | 30S ribosomal protein S19 | 1.204 | 0.26792 | 0.000935 | 0.010626 | yes | up |
| B9H01_RS01370 | B9H01_RS01370 | TetR/AcrR family transcriptional regulator | 1.199 | 0.261679 | 0.00065 | 0.008047 | yes | up |
| B9H01_RS05210 | B9H01_RS05210 | cysteine desulfurase | 1.184 | 0.243732 | 0.00181 | 0.018214 | yes | up |
| B9H01_RS01360 | B9H01_RS01360 | ABC-2 family transporter protein | 1.184 | 0.244118 | 0.001844 | 0.018435 | yes | up |
| B9H01_RS00565 | infA | translation initiation factor IF-1 | 1.182 | 0.240826 | 0.003909 | 0.033204 | yes | up |
| B9H01_RS06655 | B9H01_RS06655 | hypothetical protein | 1.177 | 0.235004 | 0.005094 | 0.040319 | yes | up |
| B9H01_RS09070 | B9H01_RS09070 | acetolactate synthase large subunit | 1.175 | 0.232175 | 0.001013 | 0.011352 | yes | up |
| B9H01_RS05800 | ptsP | phosphoenolpyruvate--protein phosphotransferase | 1.165 | 0.219863 | 5.07E-05 | 0.000873 | yes | up |
| B9H01_RS09490 | B9H01_RS09490 | ATP-dependent Clp protease ATP-binding subunit | 1.164 | 0.218874 | 0.005482 | 0.041721 | yes | up |
| B9H01_RS02070 | gpsB | cell division regulator GpsB | 1.162 | 0.216189 | 0.001321 | 0.01394 | yes | up |
| B9H01_RS08165 | B9H01_RS08165 | membrane protein | 1.162 | 0.216174 | 0.002463 | 0.023111 | yes | up |
| B9H01_RS02360 | B9H01_RS02360 | MmcQ/YjbR family DNA-binding protein | 1.159 | 0.212564 | 0.004954 | 0.039688 | yes | up |
| B9H01_RS05720 | ligA | NAD-dependent DNA ligase LigA | 1.158 | 0.211345 | 0.000534 | 0.006812 | yes | up |
| B9H01_RS05900 | B9H01_RS05900 | PTS sugar transporter subunit IIA | 1.158 | 0.212255 | 0.006455 | 0.04687 | yes | up |
| B9H01_RS09580 | rmuC | DNA recombination protein RmuC | 1.157 | 0.209953 | 0.001191 | 0.013001 | yes | up |
| B9H01_RS00460 | rplB | 50S ribosomal protein L2 | 1.152 | 0.204258 | 0.003642 | 0.031351 | yes | up |
| B9H01_RS10385 | mnmG | tRNA uridine-5-carboxymethylaminomethyl(34) synthesis enzyme MnmG | 1.152 | 0.20379 | 0.005855 | 0.043334 | yes | up |
| B9H01_RS04335 | B9H01_RS04335 | sensor histidine kinase | 1.151 | 0.202582 | 0.001797 | 0.01819 | yes | up |
| B9H01_RS09595 | rsgA | ribosome small subunit-dependent GTPase A | 1.149 | 0.200382 | 0.003948 | 0.033291 | yes | up |
| B9H01_RS00905 | rpsL | 30S ribosomal protein S12 | 1.148 | 0.198715 | 0.001053 | 0.011726 | yes | up |
| B9H01_RS09060 | ilvC | ketol-acid reductoisomerase | 1.148 | 0.199055 | 0.00623 | 0.045634 | yes | up |
| B9H01_RS09775 | radA | DNA repair protein RadA | 1.147 | 0.197483 | 0.003239 | 0.028923 | yes | up |
| B9H01_RS08160 | B9H01_RS08160 | low molecular weight phosphotyrosine protein phosphatase | 1.147 | 0.198391 | 0.003408 | 0.029797 | yes | up |
| B9H01_RS05845 | B9H01_RS05845 | LacI family DNA-binding transcriptional regulator | 1.146 | 0.196649 | 0.00238 | 0.022582 | yes | up |
| B9H01_RS09575 | B9H01_RS09575 | 3'-5' exoribonuclease YhaM family protein | 1.145 | 0.19511 | 0.004062 | 0.033919 | yes | up |
| B9H01_RS02365 | asnA | aspartate--ammonia ligase | 1.137 | 0.185791 | 0.005264 | 0.041082 | yes | up |
| B9H01_RS07975 | recN | DNA repair protein RecN | 1.136 | 0.183741 | 0.003967 | 0.033291 | yes | up |
| B9H01_RS07985 | B9H01_RS07985 | TlyA family rRNA (cytidine-2'-O)-methyltransferase | 1.131 | 0.17743 | 0.005163 | 0.040672 | yes | up |
| B9H01_RS09300 | rpsO | 30S ribosomal protein S15 | 1.128 | 0.17326 | 0.003285 | 0.029169 | yes | up |
| B9H01_RS02485 | B9H01_RS02485 | ATP-dependent Clp protease ATP-binding subunit | 1.118 | 0.161133 | 0.004219 | 0.03471 | yes | up |
| B9H01_RS02810 | B9H01_RS02810 | glutamate-5-semialdehyde dehydrogenase | 1.112 | 0.153623 | 0.004991 | 0.039688 | yes | up |
| B9H01_RS02055 | pbp1a | penicillin-binding protein PBP1A | 1.108 | 0.147322 | 0.006593 | 0.047667 | yes | up |

Note: DESeq2 software was used for difference analysis.
