# Supplementary material for: Orphan response regulator CovR plays positive regulative functions in the survivability and pathogenicity of Streptococcus suis serotype 2 isolated from a pig
Source: BMC Vet Res. 2023 Nov 22;19:243. doi: 10.1186/s12917-023-03808-9 (PMC10664645; doi:10.1186/s12917-023-03808-9)
Supplement: Supplementary file 4 — Additional file 4: Supplementary table S4. qRT-PCR validation of genes related to the pathways of fructose and mannose metabolism, glycerolipid metabolism, ABC transporters, amino sugar and nucleotide sugar metabolism and phosphotransferase system (PTS) [file 12917_2023_3808_MOESM4_ESM.docx]

Supplementary table S4 qRT-PCR validation of genes related to the pathways of fructose and mannose metabolism, glycerolipid metabolism, ABC transporters, amino sugar and nucleotide sugar metabolism and phosphotransferase system (PTS)

| **Gene ID** | **SC19** | **Δ*covR*** | **CΔ*covR*** | ***p*** |
| --- | --- | --- | --- | --- |
| **Fructose and mannose metabolism** | | | | |
| B9H01_RS08575 | 1.000±0.080^b^ | 0.737±0.084^c^ | 3.512±0.220^a^ | <0.001 |
| B9H01_RS04035 | 1.000±0.092^b^ | 0.234±0.023^c^ | 1.996±0.111^a^ | <0.001 |
| B9H01_RS04040 | 1.000±0.061^b^ | 0.387±0.075^c^ | 2.244±0.254^a^ | <0.001 |
| B9H01_RS08580 | 1.000±0.066^b^ | 0.544±0.038^c^ | 2.673±0.108^a^ | <0.001 |
| **Glycerolipid metabolism** | | | | |
| B9H01_RS07520 | 1.000±0.093^b^ | 0.002±0.000^c^ | 2.209±0.216^a^ | <0.001 |
| B9H01_RS03575 | 1.000±0.171^b^ | 0.613±0.064^c^ | 1.442±0.104^a^ | <0.001 |
| **ABC transporters** | | | | |
| B9H01_RS09200 | 1.000±0.090^b^ | 0.364±0.018^c^ | 3.333±0.198^a^ | <0.001 |
| B9H01_RS09195 | 1.000±0.113^b^ | 0.279±0.026^c^ | 2.119±0.200^a^ | <0.001 |
| B9H01_RS09190 | 1.000±0.109^b^ | 0.385±0.062^c^ | 2.742±0.218^a^ | <0.001 |
| B9H01_RS10010 | 1.000±0.110^b^ | 0.224±0.014^c^ | 2.314±0.048^a^ | <0.001 |
| B9H01_RS10265 | 1.000±0.057^b^ | 0.288±0.018^c^ | 2.442±0.214^a^ | <0.001 |
| B9H01_RS10260 | 1.000±0.167^b^ | 0.115±0.017^c^ | 2.103±0.501^a^ | <0.001 |
| B9H01_RS10025 | 1.000±0.087^b^ | 0.611±0.039^c^ | 2.845±0.295^a^ | <0.001 |
| B9H01_RS10000 | 1.000±0.083^b^ | 0.128±0.007^c^ | 1.557±0.156^a^ | <0.001 |
| B9H01_RS10255 | 1.000±0.091^b^ | 0.083±0.006^c^ | 2.394±0.277^a^ | <0.001 |
| B9H01_RS10005 | 1.000±0.114^b^ | 0.257±0.022^c^ | 1.951±0.083^a^ | <0.001 |
| **Amino sugar and nucleotide sugar metabolism** | | | | |
| B9H01_RS08575 | 1.000±0.080^b^ | 0.737±0.084^c^ | 3.512±0.220^a^ | <0.001 |
| B9H01_RS06090 | 1.000±0.101^b^ | 0.283±0.018^a^ | 2.491±0.221^a^ | <0.001 |
| B9H01_RS01845 | 1.000±0.063^b^ | 0.265±0.022^c^ | 1.975±0.124^a^ | <0.001 |
| B9H01_RS01850 | 1.000±0.088^b^ | 0.021±0.004^c^ | 2.194±0.075^a^ | <0.001 |
| B9H01_RS08580 | 1.000±0.066^b^ | 0.544±0.038^c^ | 2.673±0.108^a^ | <0.001 |
| **Phosphotransferase system** | | | | |
| B9H01_RS08575 | 1.000±0.080^b^ | 0.737±0.084^c^ | 3.512±0.220^a^ | <0.001 |
| B9H01_RS01065 | 1.000±0.091^b^ | 0.170±0.013^c^ | 2.557±0.145^a^ | <0.001 |
| B9H01_RS04035 | 1.000±0.092^b^ | 0.234±0.023^c^ | 1.996±0.111^a^ | <0.001 |
| B9H01_RS01165 | 1.000±0.068^b^ | 0.051±0.002^c^ | 2.056±0.106^a^ | <0.001 |
| B9H01_RS04040 | 1.000±0.061^b^ | 0.387±0.075^c^ | 2.244±0.254^a^ | <0.001 |
| B9H01_RS08580 | 1.000±0.066^b^ | 0.544±0.038^c^ | 2.673±0.108^a^ | <0.001 |
| B9H01_RS09285 | 1.000±0.038^b^ | 0.231±0.020^c^ | 2.680±0.114^a^ | <0.001 |

Data are means ± SD, n = 3. ^a,b,c^ Means within rows with different superscripts differ (*P* < 0.05). Differences among treatment means were determined using Duncan’s *post hoc tests*. Statistical analyses were performed using the SPSS 17.0 software (SPSS, Inc.). *P* < 0.05 was considered to indicate statistical significance.
