# Supplementary material for: Orphan response regulator CovR plays positive regulative functions in the survivability and pathogenicity of Streptococcus suis serotype 2 isolated from a pig
Source: BMC Vet Res. 2023 Nov 22;19:243. doi: 10.1186/s12917-023-03808-9 (PMC10664645; doi:10.1186/s12917-023-03808-9)
Supplement: Supplementary file 5 — Additional file 5: Supplementary table S5. The 128 areas (Identity < 85%) and 9 areas (Identity < 80%) between the genome of S. suis 2 SC19 and 05ZYH33 strains [file 12917_2023_3808_MOESM5_ESM.docx]

Supplementary table S5 The 128 areas (Identity < 85%) and 9 areas (Identity < 80%) between the genome of *S. suis* 2 SC19 and 05ZYH33 strains

| SC19 | 05ZYH33 | Identity (%) | Alignment_length | Mismatchs | Gap_openings | SC19.start | SC19.end | 05ZYH33.start | 05ZYH33.end | E-value |
| --- | --- | --- | --- | --- | --- | --- | --- | --- | --- | --- |
| CP020863.1 | CP000407.1 | 77.738 | 557 | 84 | 16 | 1713924 | 1714453 | 1713941 | 1714484 | 3.82E-80 |
| CP020863.1 | CP000407.1 | 77.738 | 557 | 84 | 16 | 1713651 | 1714194 | 1714214 | 1714743 | 3.82E-80 |
| CP020863.1 | CP000407.1 | 78.289 | 608 | 85 | 32 | 619369 | 619960 | 618748 | 619324 | 6.27E-93 |
| CP020863.1 | CP000407.1 | 78.289 | 608 | 85 | 32 | 618639 | 619215 | 619479 | 620070 | 6.27E-93 |
| CP020863.1 | CP000407.1 | 78.378 | 222 | 39 | 9 | 183901 | 184115 | 1139858 | 1139639 | 5.31E-29 |
| CP020863.1 | CP000407.1 | 78.539 | 219 | 38 | 9 | 1139421 | 1139637 | 184169 | 183958 | 5.31E-29 |
| CP020863.1 | CP000407.1 | 78.933 | 375 | 62 | 12 | 1714089 | 1714447 | 1713941 | 1714314 | 3.93E-60 |
| CP020863.1 | CP000407.1 | 78.933 | 375 | 62 | 12 | 1713651 | 1714024 | 1714379 | 1714737 | 3.93E-60 |
| CP020863.1 | CP000407.1 | 79.091 | 220 | 40 | 5 | 1380226 | 1380443 | 1572118 | 1572333 | 2.45E-32 |
| CP020863.1 | CP000407.1 | 80.319 | 188 | 29 | 7 | 1416580 | 1416761 | 58093 | 58278 | 5.31E-29 |
| CP020863.1 | CP000407.1 | 80.319 | 188 | 29 | 7 | 58092 | 58277 | 1416822 | 1417003 | 5.31E-29 |
| CP020863.1 | CP000407.1 | 81.143 | 175 | 28 | 5 | 168883 | 169053 | 1599509 | 1599682 | 5.31E-29 |
| CP020863.1 | CP000407.1 | 81.319 | 182 | 28 | 6 | 1660547 | 1660726 | 168889 | 169066 | 3.17E-31 |
| CP020863.1 | CP000407.1 | 81.752 | 137 | 22 | 3 | 2089121 | 2089256 | 500964 | 501098 | 8.95E-22 |
| CP020863.1 | CP000407.1 | 81.752 | 137 | 22 | 3 | 500858 | 500992 | 2089461 | 2089596 | 8.95E-22 |
| CP020863.1 | CP000407.1 | 81.761 | 159 | 24 | 5 | 168900 | 169053 | 1139697 | 1139855 | 8.89E-27 |
| CP020863.1 | CP000407.1 | 81.818 | 110 | 17 | 3 | 133227 | 133334 | 1544438 | 1544330 | 4.19E-15 |
| CP020863.1 | CP000407.1 | 81.977 | 172 | 31 | 0 | 501009 | 501180 | 1416910 | 1416739 | 2.45E-32 |
| CP020863.1 | CP000407.1 | 81.977 | 172 | 27 | 4 | 710240 | 710409 | 1913556 | 1913387 | 3.17E-31 |
| CP020863.1 | CP000407.1 | 81.988 | 161 | 23 | 5 | 501023 | 501179 | 1807500 | 1807658 | 6.87E-28 |
| CP020863.1 | CP000407.1 | 82.051 | 156 | 21 | 5 | 65360 | 65510 | 1775190 | 1775037 | 3.20E-26 |
| CP020863.1 | CP000407.1 | 82.09 | 134 | 21 | 3 | 1903596 | 1903728 | 501098 | 500967 | 8.95E-22 |
| CP020863.1 | CP000407.1 | 82.09 | 134 | 21 | 3 | 500861 | 500992 | 1904043 | 1903911 | 8.95E-22 |
| CP020863.1 | CP000407.1 | 82.143 | 112 | 17 | 3 | 597335 | 597445 | 338556 | 338447 | 3.24E-16 |
| CP020863.1 | CP000407.1 | 82.184 | 174 | 27 | 3 | 1416497 | 1416668 | 501286 | 501115 | 2.45E-32 |
| CP020863.1 | CP000407.1 | 82.258 | 124 | 21 | 1 | 1268041 | 1268164 | 1666520 | 1666642 | 4.16E-20 |
| CP020863.1 | CP000407.1 | 82.353 | 170 | 29 | 1 | 2089214 | 2089383 | 501114 | 501282 | 2.45E-32 |
| CP020863.1 | CP000407.1 | 82.4 | 125 | 17 | 2 | 1807060 | 1807180 | 1299762 | 1299639 | 1.50E-19 |
| CP020863.1 | CP000407.1 | 82.4 | 125 | 17 | 2 | 1299422 | 1299545 | 1807492 | 1807372 | 1.50E-19 |
| CP020863.1 | CP000407.1 | 82.456 | 171 | 27 | 3 | 501008 | 501176 | 2089554 | 2089723 | 2.45E-32 |
| CP020863.1 | CP000407.1 | 82.474 | 194 | 29 | 5 | 1380261 | 1380451 | 188588 | 188397 | 6.77E-38 |
| CP020863.1 | CP000407.1 | 82.482 | 137 | 22 | 2 | 500857 | 500992 | 1417004 | 1416869 | 5.35E-24 |
| CP020863.1 | CP000407.1 | 82.5 | 160 | 23 | 4 | 1807188 | 1807345 | 501129 | 501285 | 5.31E-29 |
| CP020863.1 | CP000407.1 | 82.53 | 166 | 25 | 4 | 1913079 | 1913242 | 710550 | 710387 | 3.17E-31 |
| CP020863.1 | CP000407.1 | 82.558 | 172 | 23 | 6 | 1870224 | 1870392 | 501286 | 501119 | 8.82E-32 |
| CP020863.1 | CP000407.1 | 82.629 | 213 | 36 | 1 | 1416530 | 1416741 | 129516 | 129304 | 1.45E-44 |
| CP020863.1 | CP000407.1 | 82.629 | 213 | 35 | 2 | 1331837 | 1332049 | 183957 | 184167 | 1.45E-44 |
| CP020863.1 | CP000407.1 | 82.71 | 214 | 34 | 3 | 129298 | 129510 | 1416983 | 1416772 | 1.45E-44 |
| CP020863.1 | CP000407.1 | 82.727 | 110 | 16 | 3 | 1544073 | 1544181 | 133342 | 133235 | 9.01E-17 |
| CP020863.1 | CP000407.1 | 82.727 | 110 | 15 | 2 | 1807074 | 1807180 | 1775206 | 1775098 | 9.01E-17 |
| CP020863.1 | CP000407.1 | 82.727 | 110 | 15 | 2 | 1774789 | 1774897 | 1807492 | 1807386 | 9.01E-17 |
| CP020863.1 | CP000407.1 | 82.787 | 122 | 20 | 1 | 338440 | 338561 | 46386 | 46266 | 1.16E-20 |
| CP020863.1 | CP000407.1 | 82.805 | 221 | 34 | 4 | 338307 | 338525 | 129517 | 129299 | 8.64E-47 |
| CP020863.1 | CP000407.1 | 82.805 | 221 | 34 | 4 | 129293 | 129511 | 338600 | 338382 | 8.64E-47 |
| CP020863.1 | CP000407.1 | 82.857 | 175 | 25 | 5 | 168883 | 169053 | 184134 | 183961 | 5.27E-34 |
| CP020863.1 | CP000407.1 | 82.877 | 146 | 25 | 0 | 1571922 | 1572067 | 139806 | 139661 | 6.87E-28 |
| CP020863.1 | CP000407.1 | 82.877 | 146 | 25 | 0 | 139653 | 139798 | 1572330 | 1572185 | 6.87E-28 |
| CP020863.1 | CP000407.1 | 82.877 | 146 | 20 | 3 | 1774737 | 1774881 | 65502 | 65361 | 3.20E-26 |
| CP020863.1 | CP000407.1 | 82.927 | 123 | 16 | 2 | 1299422 | 1299543 | 338515 | 338633 | 4.16E-20 |
| CP020863.1 | CP000407.1 | 82.927 | 123 | 16 | 2 | 338440 | 338558 | 1299639 | 1299760 | 4.16E-20 |
| CP020863.1 | CP000407.1 | 82.967 | 182 | 28 | 3 | 658866 | 659046 | 129342 | 129521 | 8.76E-37 |
| CP020863.1 | CP000407.1 | 82.967 | 182 | 28 | 3 | 129336 | 129515 | 658987 | 659167 | 8.76E-37 |
| CP020863.1 | CP000407.1 | 82.979 | 235 | 34 | 6 | 1443693 | 1443923 | 129294 | 129526 | 1.11E-50 |
| CP020863.1 | CP000407.1 | 82.979 | 235 | 34 | 6 | 129288 | 129520 | 1443940 | 1444170 | 1.11E-50 |
| CP020863.1 | CP000407.1 | 83.146 | 178 | 26 | 3 | 58091 | 58268 | 129467 | 129294 | 3.15E-36 |
| CP020863.1 | CP000407.1 | 83.193 | 119 | 19 | 1 | 46269 | 46386 | 338633 | 338515 | 1.16E-20 |
| CP020863.1 | CP000407.1 | 83.255 | 424 | 70 | 1 | 1312628 | 1313050 | 1312664 | 1313087 | 3.69E-105 |
| CP020863.1 | CP000407.1 | 83.294 | 419 | 70 | 0 | 1312452 | 1312870 | 1312849 | 1313267 | 1.33E-104 |
| CP020863.1 | CP000407.1 | 83.333 | 156 | 24 | 2 | 1380298 | 1380452 | 492667 | 492513 | 3.17E-31 |
| CP020863.1 | CP000407.1 | 83.333 | 96 | 10 | 5 | 262136 | 262228 | 262002 | 262094 | 1.95E-13 |
| CP020863.1 | CP000407.1 | 83.333 | 96 | 10 | 5 | 261926 | 262018 | 262212 | 262304 | 1.95E-13 |
| CP020863.1 | CP000407.1 | 83.429 | 175 | 25 | 3 | 129291 | 129461 | 58266 | 58092 | 3.15E-36 |
| CP020863.1 | CP000407.1 | 83.486 | 218 | 33 | 3 | 356333 | 356549 | 129521 | 129306 | 1.86E-48 |
| CP020863.1 | CP000407.1 | 83.486 | 218 | 33 | 3 | 129300 | 129515 | 356626 | 356410 | 1.86E-48 |
| CP020863.1 | CP000407.1 | 83.486 | 218 | 29 | 7 | 2052209 | 2052424 | 1697786 | 1697998 | 2.40E-47 |
| CP020863.1 | CP000407.1 | 83.486 | 218 | 29 | 7 | 1697499 | 1697711 | 2052539 | 2052754 | 2.40E-47 |
| CP020863.1 | CP000407.1 | 83.495 | 103 | 12 | 5 | 1903650 | 1903748 | 1946915 | 1947016 | 1.17E-15 |
| CP020863.1 | CP000407.1 | 83.523 | 176 | 22 | 6 | 168883 | 169053 | 1660822 | 1660995 | 1.13E-35 |
| CP020863.1 | CP000407.1 | 83.537 | 164 | 18 | 8 | 501022 | 501180 | 1870698 | 1870539 | 8.82E-32 |
| CP020863.1 | CP000407.1 | 83.588 | 262 | 39 | 4 | 1903467 | 1903727 | 1416741 | 1416999 | 3.04E-61 |
| CP020863.1 | CP000407.1 | 83.588 | 262 | 39 | 4 | 1416499 | 1416757 | 1903782 | 1904042 | 3.04E-61 |
| CP020863.1 | CP000407.1 | 83.594 | 128 | 19 | 2 | 1416636 | 1416762 | 501089 | 500963 | 5.35E-24 |
| CP020863.1 | CP000407.1 | 83.696 | 184 | 25 | 4 | 356388 | 356567 | 58093 | 58275 | 5.24E-39 |
| CP020863.1 | CP000407.1 | 83.696 | 184 | 25 | 4 | 58092 | 58274 | 356465 | 356644 | 5.24E-39 |
| CP020863.1 | CP000407.1 | 83.761 | 117 | 15 | 4 | 597335 | 597450 | 1840571 | 1840684 | 1.16E-20 |
| CP020863.1 | CP000407.1 | 83.81 | 105 | 15 | 2 | 1903646 | 1903748 | 1156901 | 1156797 | 6.97E-18 |
| CP020863.1 | CP000407.1 | 83.81 | 105 | 15 | 2 | 1156601 | 1156705 | 1904063 | 1903961 | 6.97E-18 |
| CP020863.1 | CP000407.1 | 83.846 | 130 | 18 | 3 | 500865 | 500992 | 338608 | 338480 | 1.49E-24 |
| CP020863.1 | CP000407.1 | 83.846 | 130 | 18 | 3 | 338405 | 338533 | 501098 | 500971 | 1.49E-24 |
| CP020863.1 | CP000407.1 | 83.871 | 93 | 14 | 1 | 1840203 | 1840295 | 65359 | 65450 | 1.51E-14 |
| CP020863.1 | CP000407.1 | 83.871 | 93 | 14 | 1 | 65358 | 65449 | 1840520 | 1840612 | 1.51E-14 |
| CP020863.1 | CP000407.1 | 83.916 | 143 | 20 | 3 | 1339176 | 1339316 | 151141 | 151000 | 1.91E-28 |
| CP020863.1 | CP000407.1 | 83.916 | 143 | 20 | 3 | 150995 | 151136 | 1339540 | 1339400 | 1.91E-28 |
| CP020863.1 | CP000407.1 | 83.942 | 137 | 20 | 2 | 683869 | 684004 | 65366 | 65501 | 2.47E-27 |
| CP020863.1 | CP000407.1 | 83.942 | 137 | 20 | 2 | 65365 | 65500 | 684014 | 684149 | 2.47E-27 |
| CP020863.1 | CP000407.1 | 83.978 | 181 | 21 | 4 | 1299369 | 1299548 | 46436 | 46263 | 1.88E-38 |
| CP020863.1 | CP000407.1 | 83.978 | 181 | 21 | 4 | 46263 | 46436 | 1299765 | 1299586 | 1.88E-38 |
| CP020863.1 | CP000407.1 | 84.021 | 194 | 25 | 5 | 338357 | 338546 | 58092 | 58283 | 6.72E-43 |
| CP020863.1 | CP000407.1 | 84.021 | 194 | 25 | 5 | 58091 | 58282 | 338432 | 338621 | 6.72E-43 |
| CP020863.1 | CP000407.1 | 84.043 | 94 | 11 | 2 | 684134 | 684224 | 1775310 | 1775218 | 1.51E-14 |
| CP020863.1 | CP000407.1 | 84.091 | 132 | 18 | 3 | 500863 | 500992 | 356643 | 356513 | 1.15E-25 |
| CP020863.1 | CP000407.1 | 84.091 | 132 | 18 | 3 | 356436 | 356566 | 501098 | 500969 | 1.15E-25 |
| CP020863.1 | CP000407.1 | 84.099 | 283 | 39 | 6 | 1807072 | 1807352 | 1904053 | 1903775 | 5.02E-69 |
| CP020863.1 | CP000407.1 | 84.118 | 170 | 25 | 2 | 356304 | 356473 | 501286 | 501119 | 2.44E-37 |
| CP020863.1 | CP000407.1 | 84.173 | 139 | 22 | 0 | 492420 | 492558 | 139661 | 139799 | 5.31E-29 |
| CP020863.1 | CP000407.1 | 84.173 | 139 | 22 | 0 | 139653 | 139791 | 492525 | 492663 | 5.31E-29 |
| CP020863.1 | CP000407.1 | 84.186 | 215 | 34 | 0 | 1571856 | 1572070 | 1350574 | 1350788 | 3.08E-51 |
| CP020863.1 | CP000407.1 | 84.186 | 215 | 34 | 0 | 1350337 | 1350551 | 1572119 | 1572333 | 3.08E-51 |
| CP020863.1 | CP000407.1 | 84.302 | 172 | 21 | 4 | 1774738 | 1774906 | 1946845 | 1947013 | 2.44E-37 |
| CP020863.1 | CP000407.1 | 84.314 | 102 | 12 | 4 | 1946594 | 1946694 | 1903965 | 1904063 | 2.51E-17 |
| CP020863.1 | CP000407.1 | 84.375 | 128 | 16 | 3 | 1870308 | 1870431 | 58093 | 58220 | 4.13E-25 |
| CP020863.1 | CP000407.1 | 84.375 | 128 | 16 | 3 | 58092 | 58219 | 1870623 | 1870746 | 4.13E-25 |
| CP020863.1 | CP000407.1 | 84.387 | 269 | 37 | 5 | 1416497 | 1416761 | 338348 | 338615 | 3.02E-66 |
| CP020863.1 | CP000407.1 | 84.387 | 269 | 37 | 5 | 338273 | 338540 | 1416739 | 1417003 | 3.02E-66 |
| CP020863.1 | CP000407.1 | 84.397 | 141 | 17 | 4 | 139654 | 139791 | 1380678 | 1380540 | 1.91E-28 |
| CP020863.1 | CP000407.1 | 84.404 | 109 | 17 | 0 | 1268057 | 1268165 | 356264 | 356156 | 1.16E-20 |
| CP020863.1 | CP000407.1 | 84.404 | 109 | 17 | 0 | 356079 | 356187 | 1268377 | 1268269 | 1.16E-20 |
| CP020863.1 | CP000407.1 | 84.404 | 109 | 16 | 1 | 500880 | 500987 | 129306 | 129414 | 4.16E-20 |
| CP020863.1 | CP000407.1 | 84.404 | 109 | 16 | 1 | 129300 | 129408 | 500986 | 501093 | 4.16E-20 |
| CP020863.1 | CP000407.1 | 84.434 | 212 | 33 | 0 | 1599207 | 1599418 | 184169 | 183958 | 3.08E-51 |
| CP020863.1 | CP000407.1 | 84.434 | 212 | 33 | 0 | 183901 | 184112 | 1599685 | 1599474 | 3.08E-51 |
| CP020863.1 | CP000407.1 | 84.536 | 194 | 23 | 6 | 1903548 | 1903736 | 58092 | 58283 | 5.20E-44 |
| CP020863.1 | CP000407.1 | 84.536 | 194 | 23 | 6 | 58091 | 58282 | 1903863 | 1904051 | 5.20E-44 |
| CP020863.1 | CP000407.1 | 84.545 | 220 | 27 | 6 | 1660510 | 1660723 | 1139640 | 1139858 | 8.58E-52 |
| CP020863.1 | CP000407.1 | 84.545 | 220 | 27 | 6 | 1139419 | 1139637 | 1660785 | 1660998 | 8.58E-52 |
| CP020863.1 | CP000407.1 | 84.571 | 175 | 23 | 2 | 1299371 | 1299544 | 1946845 | 1947016 | 1.46E-39 |
| CP020863.1 | CP000407.1 | 84.615 | 182 | 14 | 8 | 1913072 | 1913240 | 1014813 | 1014993 | 5.24E-39 |
| CP020863.1 | CP000407.1 | 84.615 | 182 | 14 | 8 | 1014622 | 1014802 | 1913386 | 1913554 | 5.24E-39 |
| CP020863.1 | CP000407.1 | 84.615 | 143 | 20 | 2 | 1799319 | 1799460 | 65502 | 65361 | 1.14E-30 |
| CP020863.1 | CP000407.1 | 84.615 | 143 | 20 | 2 | 65360 | 65501 | 1799771 | 1799630 | 1.14E-30 |
| CP020863.1 | CP000407.1 | 84.615 | 130 | 17 | 3 | 1298690 | 1298818 | 357992 | 358119 | 3.20E-26 |
| CP020863.1 | CP000407.1 | 84.615 | 130 | 17 | 3 | 357915 | 358042 | 1298907 | 1299035 | 3.20E-26 |
| CP020863.1 | CP000407.1 | 84.615 | 91 | 10 | 2 | 1774912 | 1775001 | 684366 | 684279 | 1.51E-14 |
| CP020863.1 | CP000407.1 | 84.791 | 263 | 37 | 3 | 1416497 | 1416756 | 356381 | 356643 | 8.40E-67 |
| CP020863.1 | CP000407.1 | 84.791 | 263 | 37 | 3 | 356304 | 356566 | 1416739 | 1416998 | 8.40E-67 |
| CP020863.1 | CP000407.1 | 84.795 | 171 | 21 | 3 | 1946524 | 1946691 | 1775047 | 1775215 | 1.88E-38 |
| CP020863.1 | CP000407.1 | 84.848 | 165 | 21 | 4 | 501022 | 501183 | 356541 | 356378 | 2.44E-37 |
| CP020863.1 | CP000407.1 | 84.884 | 86 | 13 | 0 | 139742 | 139827 | 217888 | 217803 | 1.51E-14 |
