# Supplementary material for: Orphan response regulator CovR plays positive regulative functions in the survivability and pathogenicity of Streptococcus suis serotype 2 isolated from a pig
Source: BMC Vet Res. 2023 Nov 22;19:243. doi: 10.1186/s12917-023-03808-9 (PMC10664645; doi:10.1186/s12917-023-03808-9)
Supplement: Supplementary file 6 — Additional file 6: Figure S1. The identification of the knockout mutant ΔcovR and complementation strain CΔcovR. PCR confirmation of the mutant and CΔcovR. The primer pairs (the forward primer 5′-TCAATCGCGCATGGC-3′ and the reverse primer 5′-ATGGCTAAGAAAATTTTGATTG-3′ were used in the PCR, the 500 bp DNA fragment was amplified by using the primer pairs. Lane M indicated the DL5000 DNA Marker, lane 1 indicated the negative control without template, templates were genomic DNA from ΔcovR (lane 2), CΔcovR (lane 3), and S.suis 2 SC19 (lane 4). Figure S2 The number of genes expressed in SC19 and ΔcovR. A The Venn diagram showed 1823 genes are expressed inSC19 (Light red) and ΔcovR (watchet). B Principal component analysis (PCA) of RNA-Seq data. The percentages on each axis represent the percentages of variation explained by the principal components. Points that are closer together are more similar in gene expression patterns. Figure S3 Differentially expressed genes of ΔcovR compared to SC19. a Volcano plot showing the fold change (log2 ratio) in the expression of differentially expressed genes in ΔcovR vs. SC19 (X-axis) plotted against the -log10 adjusted p-value (Y-axis). Each red square, gray dot and blue triangle on the plot represents the mean value (from three independent cultures) of one gene. Red square: Significantly up-regulated genes. Blue triangle: Significantly down-regulated genes. Gray dot: no significant difference genes. Figure S4 The GO andKEGG analysis of 114 down-regulated Genes. A GO annotations analysis. B KEGG analysis. Figure S5 The GO annotations analysis of 117 up-regulated Genes. Figure S6 The collinearity analysis of S. suis 2 SC19 and 05ZYH33 strains. The genomes of S. suis 2 SC19 and 05ZYH33 strains are similar, however, the location and copy numbers of many gene elements in the genome were changed between S. suis 2 SC19 and 05ZYH33 strains [file 12917_2023_3808_MOESM6_ESM.docx]

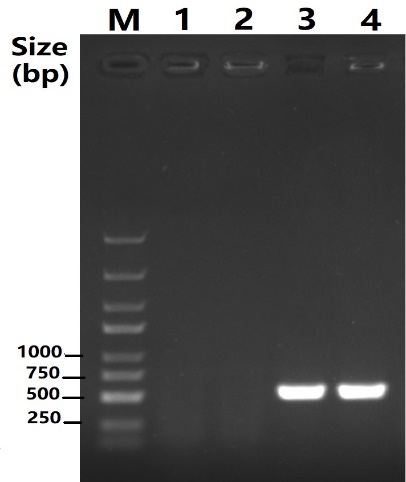


**Figure S1 The identification of the knockout mutant** Δ***covR* and complementation strain C**Δ***covR*.** PCR confirmation of the mutant and CΔ*covR*. The primer pairs (the forward primer 5′-TCAATCGCGCATGGC-3′ and the reverse primer 5′-ATGGCTAAGAAAATTTTGATTG-3′ were used in the PCR, the 500 bp DNA fragment was amplified by using the primer pairs. Lane M indicated the DL5000 DNA Marker, lane 1 indicated the negative control without template, templates were genomic DNA from Δ*covR* (lane 2), CΔ*covR* (lane 3), and *S.suis* 2 SC19 (lane 4).


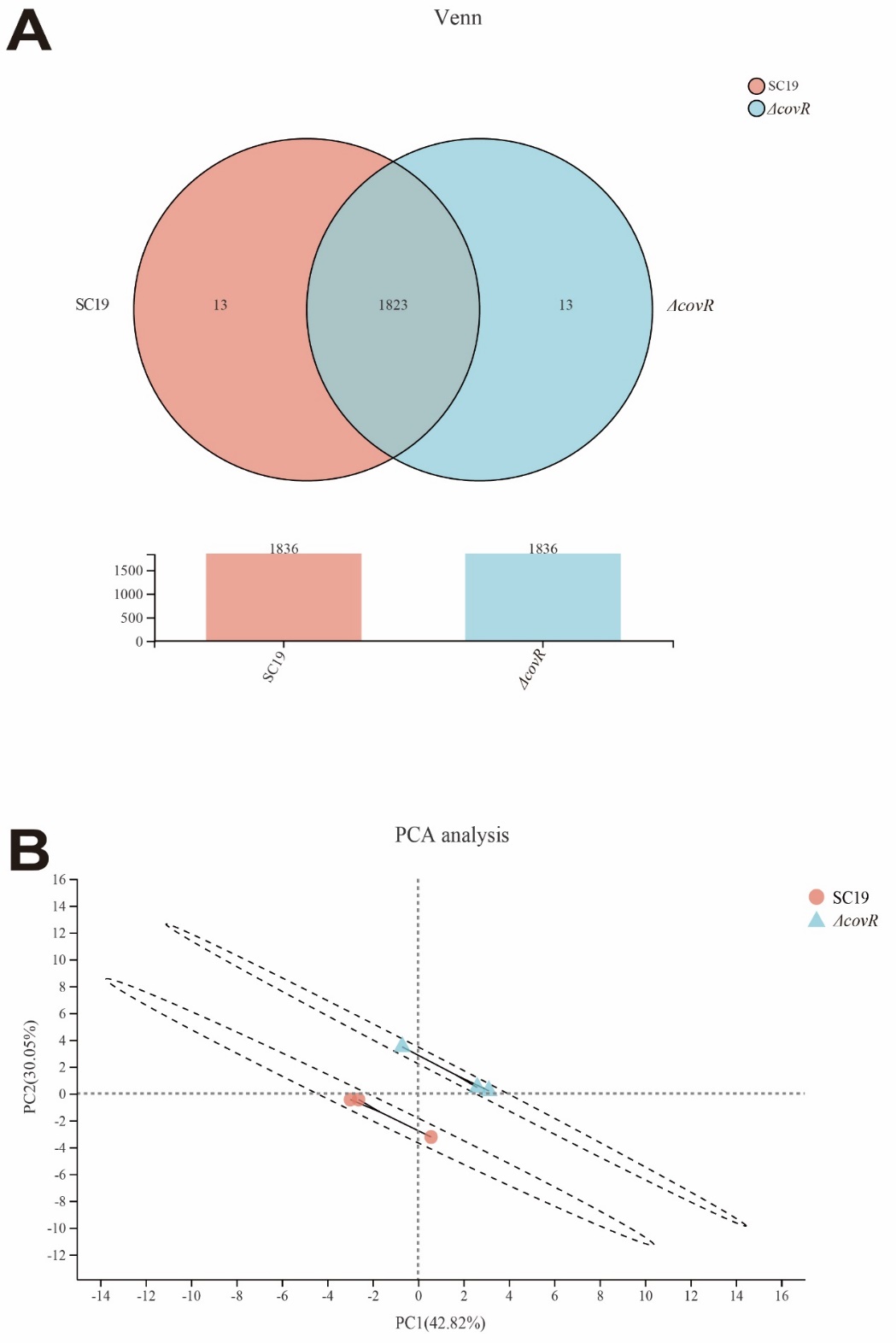


**Figure S2** The number of genes expressed in SC19 and Δ*covR*. **A** The Venn diagram showed 1823 genes are expressed in SC19 (Light red) and Δ*covR* (watchet). **B** Principal component analysis (PCA) of RNA-Seq data. The percentages on each axis represent the percentages of variation explained by the principal components. Points that are closer together are more similar in gene expression patterns.


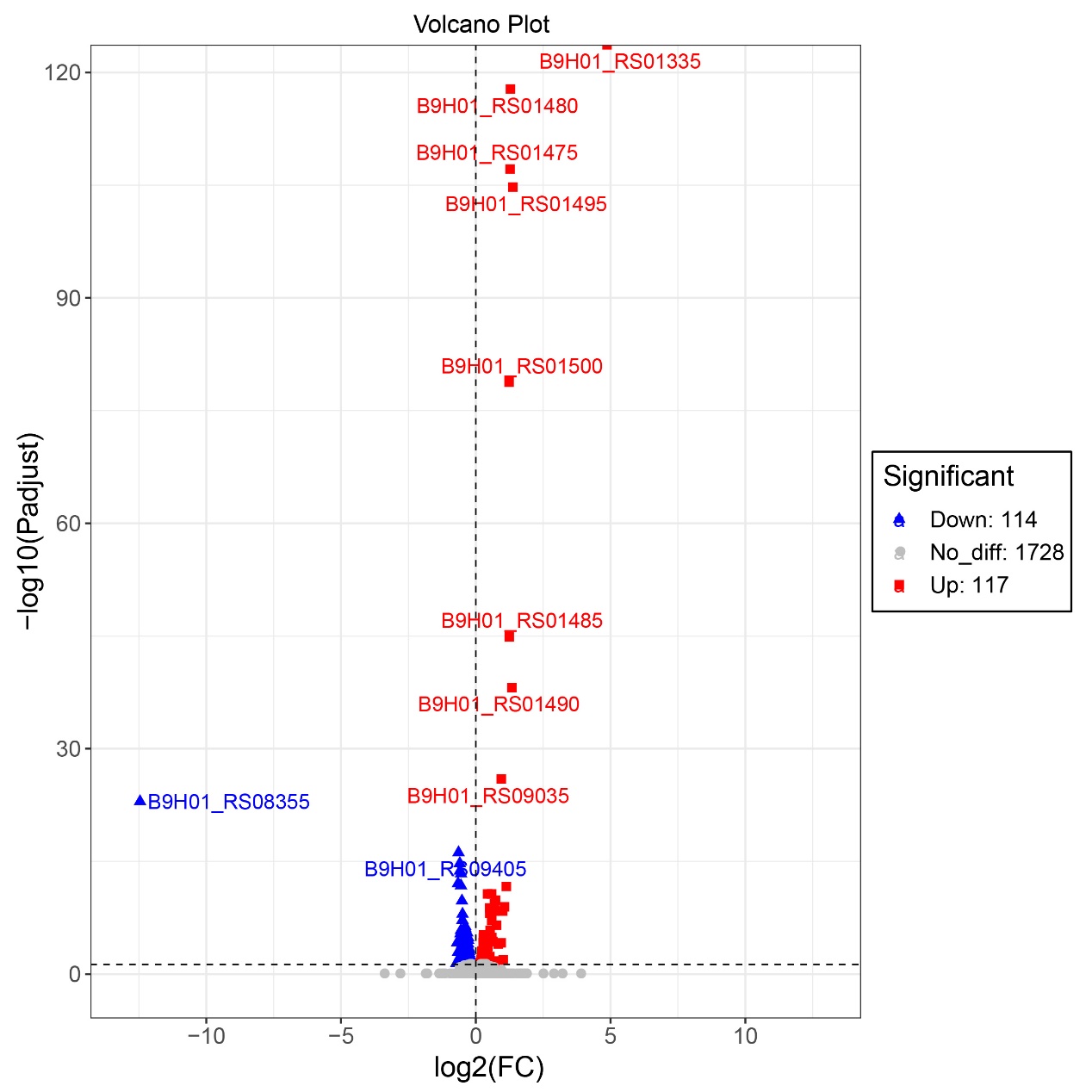


**Figure S3** Differentially expressed genes of Δ*covR* compared to SC19. a Volcano plot showing the fold change (log2 ratio) in the expression of differentially expressed genes in Δ*covR* vs. SC19 (X-axis) plotted against the -log_10_ adjusted p-value (Y-axis). Each red square, gray dot and blue triangle on the plot represents the mean value (from three independent cultures) of one gene. Red square: Significantly up-regulated genes. Blue triangle: Significantly down-regulated genes. Gray dot: no significant difference genes.


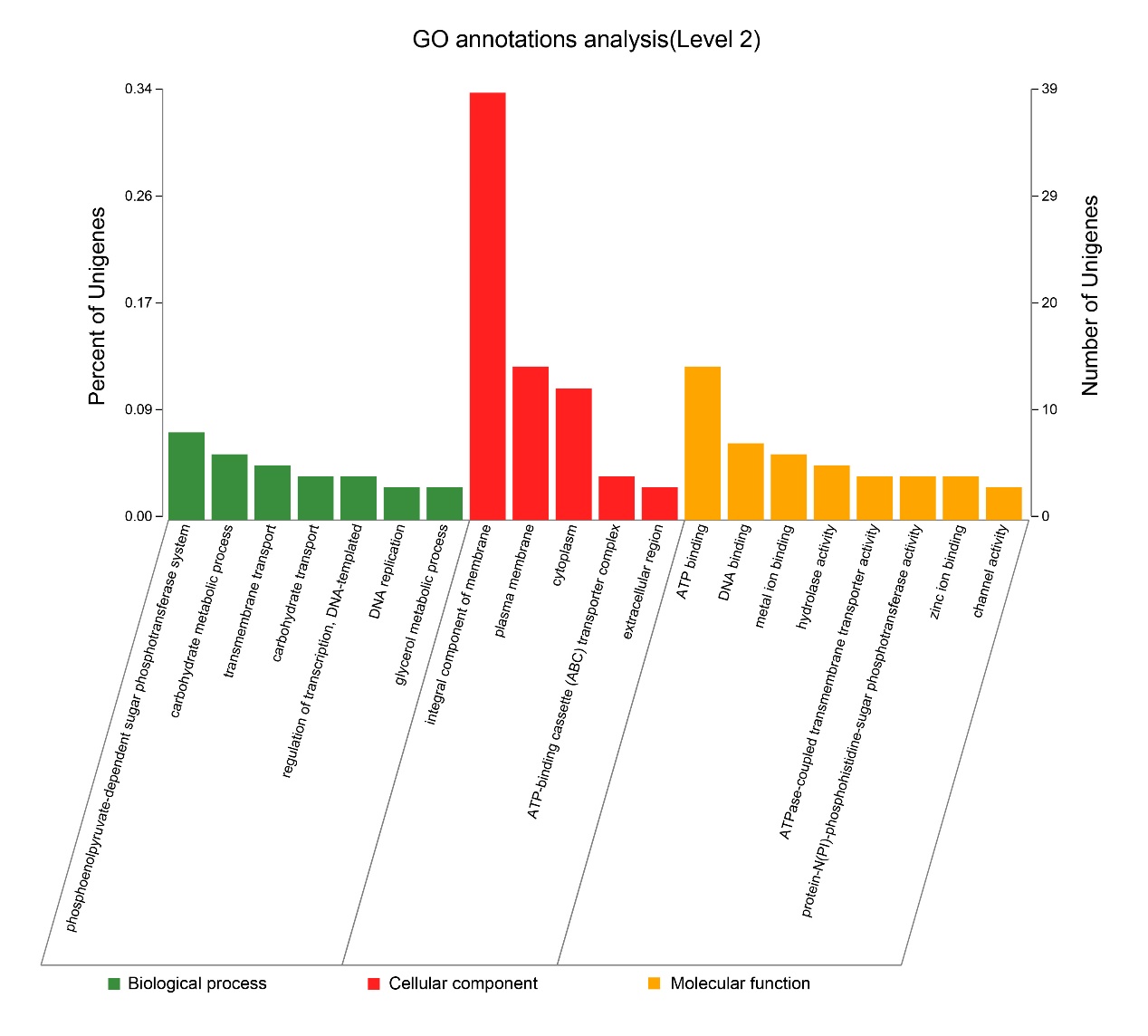


**A**


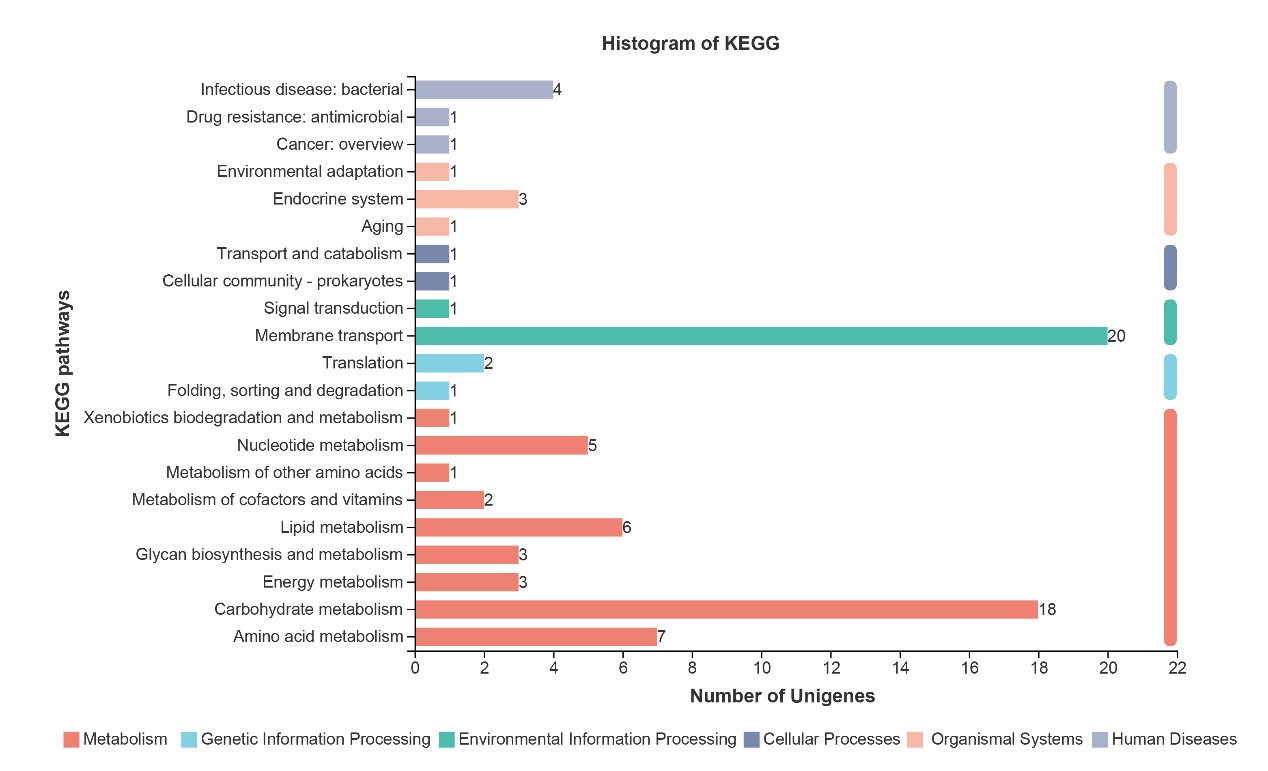
 **Figure S4** The GO and KEGG analysis of 114 down-regulated Genes. **A G**O annotations analysis. **B** KEGG analysis.

**B**


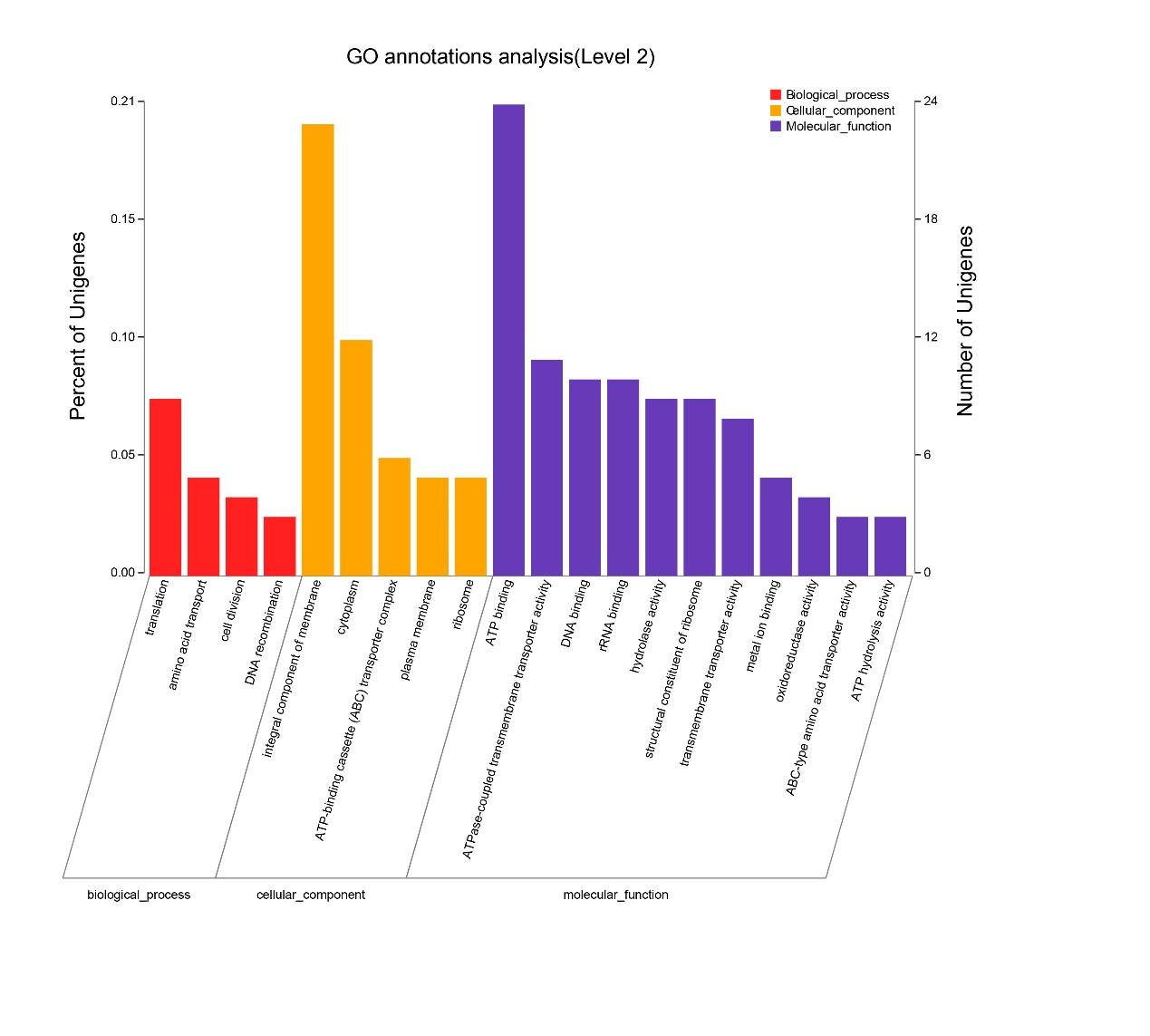


**Figure S5** The GO annotations analysis of 117 up-regulated Genes.


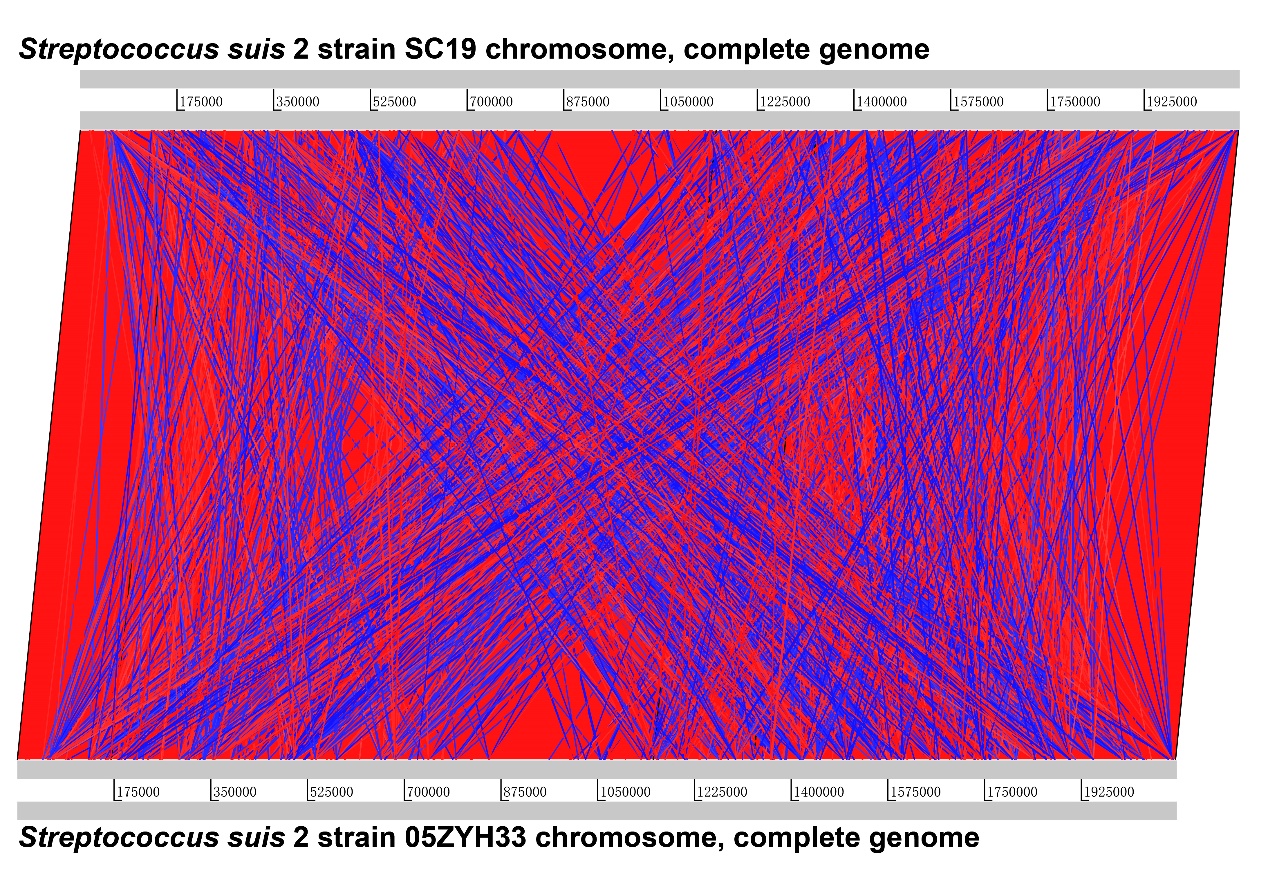


**Figure S6** The collinearity analysis of *S. suis* 2 SC19 and 05ZYH33 strains. The genomes of *S. suis* 2 SC19 and 05ZYH33 strains are similar, however, the location and copy numbers of many gene elements in the genome were changed between *S. suis* 2 SC19 and 05ZYH33 strains.
